# Construction and characterization of bacterial artificial chromosomes harboring the full-length genome of a highly attenuated vaccinia virus LC16m8

**DOI:** 10.1371/journal.pone.0192725

**Published:** 2018-02-23

**Authors:** Tomoki Yoshikawa, Hikaru Fujii, Akiko Okutani, Miho Shibamura, Natsumi Omura, Kazutaka Egawa, Hirofumi Kato, Takuya Inagaki, Shizuko Harada, Souichi Yamada, Shigeru Morikawa, Masayuki Saijo

**Affiliations:** 1 Department of Virology 1, National Institute of Infectious Diseases, Toyama, Shinjuku-ku, Tokyo, Japan; 2 Department of Veterinary Science, National Institute of Infectious Diseases, Toyama, Shinjuku-ku, Tokyo, Japan; 3 Department of Life Science and Medical Bioscience, Waseda University, Wakamatsu-cho, Shinjuku-ku, Tokyo, Japan; Keck School of Medicine of the University of Southern California, UNITED STATES

## Abstract

LC16m8 (m8), a highly attenuated vaccinia virus (VAC) strain, was developed as a smallpox vaccine, and its safety and immunogenicity have been confirmed. Here, we aimed to develop a system that recovers infectious m8 from a bacterial artificial chromosome (BAC) that retains the full-length viral genomic DNA (m8-BAC system). The infectious virus was successfully recovered from a VAC-BAC plasmid, named pLC16m8-BAC. Furthermore, the bacterial replicon-free virus was generated by intramolecular homologous recombination and was successfully recovered from a modified VAC-BAC plasmid, named pLC16m8.8S-BAC. Also, the growth of the recovered virus was indistinguishable from that of authentic m8. The full genome sequence of the plasmid, which harbors identical inverted terminal repeats (ITR) to that of authentic m8, was determined by long-read next-generation sequencing (NGS). The ITR contains x 18 to 32 of the 70 and x 30 to 45 of 54 base pair tandem repeats, and the number of tandem repeats was different between the ITR left and right. Since the virus recovered from pLC16m8.8S-BAC was expected to retain the identical viral genome to that of m8, including the ITR, a reference-based alignment following a short-read NGS was performed to validate the sequence of the recovered virus. Based on the pattern of coverage depth in the ITR, no remarkable differences were observed between the virus and m8, and the other region was confirmed to be identical as well. In summary, this new system can recover the virus, which is geno- and phenotypically indistinguishable from authentic m8.

## Introduction

The virulence and immunogenicity of each vaccinia virus (VAC) strain are highly diverse. Some strains, such as VAC, Lister, and New York City Board of Health strains (NYCBH) that were used for the smallpox eradication program caused serious adverse events, such as postvaccinial encephalitis, [1, 2]. On the other hand, a VAC strain, LC16m8 (m8), was found to be less virulent and safer than the other vaccinia strains when evaluated in different ways, including intrathalamic inoculation into cynomolgus monkeys, proliferation in rabbit skin, and immunization of 90,000 infants as a clinical study [2, 3]. Furthermore, the immunogenicity and efficacy of m8 as a vaccine relative to that of other orthopoxviral diseases, such as monkeypox in nonhuman primates, are similar to that of the other VAC strains [4–9]. After the eradication of smallpox, VAC strains were still expected as the vaccine for smallpox, which has potential use as a bioterrorism agent and also as a recombinant vaccine vector. Highly attenuated VAC strains are naturally the first choice for these uses.

In fact, modified vaccinia Ankara (MVA) [10–12], a highly attenuated smallpox vaccine, has been actively studied for its potential as a recombinant vaccine vector against malaria, influenza, ebola, etc. [13–15]. In contrast, m8 has been less well studied even though it has similar safety and immunogenicity parameters as MVA. One reason for the focus on MVA is that it is a convenient system to make recombinant VAC, such as the VAC-bacterial artificial chromosome (VAC-BAC) system [16, 17] that was established based on MVA [18, 19]. In contrast, no such system has yet been developed based on m8. The VAC-BAC system is used to recover the infectious VAC from a VAC-BAC plasmid, which harbors the full-length VAC genome with a regulatory element, such as mini-F cassette for stably maintaining the plasmid in *E*. *coli*. Although the system requires infection with the plasmid and fowlpox virus as a helper virus, the VAC genome in the plasmid can be genetically modified quickly and easily on demand by *E*. *coli* genetics, and the recombinant VAC can be generated from the modified VAC-BAC plasmid. Therefore, in this study, we aimed to establish the VAC-BAC system based on the m8 (m8-BAC system).

This m8-BAC system was constructed to allow recovery of the virus that was geno- and phenotypically indistinguishable to authentic m8 by self-excision of a mini-F cassette. Since the sequence of the inverted terminal repetitions (ITR), which is a characteristic of the poxviral genome [20], was not determined in the m8 genome, full-genome sequencing of the established BAC plasmid, including the ITRs, was performed by long-read next-generation sequencing (NGS) technologies. Also, a short-read NGS was performed to validate the sequence of the recovered virus, especially the ITRs, in comparison with that of m8 based on the pattern of coverage depth in the ITR.

## Materials and methods

### Cells and viruses

293FT cells (Thermo Fisher Scientific, Waltham, MA) and RK13 cells were grown in Dulbecco's Modified Eagle Medium (DMEM; WAKO, Osaka, Japan) supplemented with 5% heat-inactivated fetal bovine serum (FBS) (DMEM-5FBS). The VAC strains LC16m8 (m8) (DDBJ/EMBL/GenBank accession no. AY678275) and LC16mO (mO, accession no. AY678277, a kind gift from Dr. So Hashizume) were propagated and titrated by the standard plaque assay on RK13 cells [5]. The fowlpox virus strain Beaudette was propagated and titrated on chicken embryo fibroblast cells. The virus titration was based on the standard plaque assay with visualization of the plaques with 100-fold diluted chicken anti-fowlpox antibody (MyBioSource, Vancouver, Canada) followed by 1.5 μg/ml of Pierce FITC-conjugated rabbit anti-chicken IgY (Thermo Fisher Scientific).

### Plasmids

The Plasmids, named pVAC-T7pol-Cas9.1 (accession no. LC214748), pVAC-BAC11 (accession no. LC214749) and pUC-Zeo-S, were constructed as follows. pVAC-T7pol-Cas9.1 harbors bacteriophage T7 polymerase and Cas9, an RNA-guided DNA endonuclease expression cassettes, and both of the open reading frames are driven by a synthetic VAC early/late promoter [21]. pVAC-BAC11 harbored a mini-F cassette, which originated at the sequence position 646 to 7030 in a pBeloBAC11 plasmid (New England BioLabs, Ipswich, Massachusetts) and an EGFP expression cassette, which was driven by the synthetic VAC early/late promoter. These mini-F and EGFP cassettes were flanked by the front (sequence position of 170889 to 171287) and rear (171311 to 171710) regions around the m8 B5R gene. The pVAC-BAC11 also harbored a guide RNA (gRNA) scaffold, which was driven by the T7 promoter. The target gRNA sequence (TTGCAACTTAGTGTCATGG), which was identical to a part of the m8 B5R gene (reversely complemented nucleotide position 171291 to 171309), was inserted into the gRNA cassette by the BbsI restriction enzyme. pEPkan-S harbored a site, which was recognized by a restriction endonuclease, I-SceI, and a kanamycin-resistance cassette. The pEPkan-S was kindly provided by Dr. Yasushi Kawaguchi at The University of Tokyo with the permission of Dr. Nikolaus Osterrieder at the Free University of Berlin, Germany [22, 23]. pUC-Zeo harbored a site, which was recognized by the I-SceI restriction endonuclease, and a Zeocin resistance cassette.

### Construction of a recombinant m8 harboring EGFP and mini-F cassettes

A CRISPR-Cas9 system for the VAC [24] was adopted with some modifications for constructing a recombinant m8, which harbored a mini-F and an EGFP cassette (m8-EGFP-BAC). First, 2.5 x 10^5^ 293FT cells were transfected with the 0.25 μg of pVAC-BAC11 and 0.25 μg of pVAC-T7pol-Cas9.1 with 1.5 μl of FuGENE 6 (Promega, Fitchburg, WI) immediately before the infection with the m8 at a multiplicity of infection (MOI) of 0.05 per cell. The pVAC-BAC11, which was used for the transfection, was NotI digested where the site was located immediately beneath the gRNA scaffold. The cells infected with m8 along with DMEM-5FBS were collected at 3 day post infection (d.p.i.) and were freeze-thawed three times to prepare a crude m8-EGFP-BAC stock. RK13 cells in monolayer culture were infected with the m8-EGFP-BAC stock, and the cells were overlaid with DMEM containing 2% FBS (DMEM-2FBS) plus 1% agarose ME (Iwai Chemicals, Tokyo, Japan). At 3 d.p.i., the EGFP fluorescence-positive plaques were visualized on a LED transilluminator (GELmieru, WAKO) at a 500 nm wavelength. The m8-EGFP-BAC was cloned by repeating the cycle of infection and plaque picking twice. A conventional PCR was performed to confirm the presence of the virus harboring the mini-F cassette. The PCR template was prepared as follows. First, 8.5 μl of m8-EGFP-BAC stock solution (approximately 10^6^ to 10^7^ pfu/ml of viruses in DMEM-5 FBS) was mixed with 1 μl of 10 x Proteinase K lysis buffer (100 mM Tris-Cl; pH 8.0, 4.5% NP40, and 4.5% Tween 20) and 0.5 μl of 20 mg/ml proteinase K (Takara, Shiga, Japan). After incubation at 56°C for 30 min and at 95°C for 5 min, 1 μl of the proteinase K treated solution was added as the template to 9 μl of PCR reaction mixtures of SapphireAmp Fast PCR master mix (Takara), with contains 1 x master mix, H_2_O, and 200 nM of a specific primer set, which targets to the *repE* gene in the mini-F cassette (Forward; 5'-AGAGGATGCCGGCGATGAAA-3', reverse; 5'-GAGGCGCATTGGAGTTCTGC-3'). Forty cycles of amplification under the following conditions: denaturing at 98°C for 5 sec, annealing at 60°C for 5 sec, and extension at 72°C for 10 sec, followed by a 1°C decrease in the annealing temperature during the first five cycles and maintenance of the annealing at 55°C for the rest of the cycles. After electrophoresis on 1% agarose gels, the PCR products with expected sizes (373 bp) were visualized by staining with GelRed (Biotium, Hayward, CA).

### Construction of a VAC-BAC plasmid harboring the full-length m8 genome

The procedure to generate a VAC-BAC plasmid harboring the full-length m8 genome (pLC16m8-BAC) was adopted based on previous studies [16–18, 25–27]. RK13 cells were infected with m8-EGFP-BAC at an MOI of 3 followed by the immediate addition of 15 μM of isatin-beta-thiosemicarbazone (IβT; CAS no.487-16-1, Frinton Laboratories, Hainesport, NJ). At 1 d.p.i., the extrachromosomal DNA was purified from the infected cells by the Hirt’s extraction protocol [27, 28]. An electrocompetent *E*. *coli*, HST08 premium electro cells (Takara), were electroporated with the extrachromosomal DNA at 200 Ω, 25 μF, and 1500 V by the Gene Pulser II (BioRad, Munchen, Germany). The cells were then cultivated on LB agar plates for two days with 20 μg/ml of chloramphenicol selective pressure. Each of the chloramphenicol-resistant *E*. *coli* colonies was selected and cultured in 2 ml of LB medium with 20 μg/ml of chloramphenicol. The plasmid was purified from each culture by an alkaline lysis method, and conventional multiplex PCR was used to confirm the presence of the m8 genome in the plasmid.

### Mutagenesis of pLC16m8-BAC

A two-step markerless Red recombination system was adopted to construct the VAC-BAC plasmids, pLC16m8.O-BAC, pLC16m8.8-BAC, pLC16m8.OS-BAC, and pLC16m8.8S-BAC [22, 23]. An *E*. *coli* strain GS1783 that encodes inducible Red- and I-SceI-expression in the chromosome, a kind gift from Dr. Yasushi Kawaguchi at The University of Tokyo, Japan with permission for use by Dr. Gregory A Smith at Northwestern University, USA [23] was used. Briefly, the GS1783 was electroporated with pLC16m8-BAC (GS1783-m8-BAC) and then cloned with 20 μg/ml of chloramphenicol selective pressure for construction of pLC16m8.OS-BAC, and pLC16m8.8S-BAC. Electrocompetent cells were prepared from the GS1783-m8-BAC with induction of the Red recombination system by incubation at 42°C for 15 min. Next, a PCR product, which was combined the rear part of B5R gene with an antibiotic-resistant gene against kanamycin, I-SceI site was prepared by overlap extension PCR [29] using two PCR products amplified from templates either pEPkan-S or a plasmid harboring the B5R gene. The PCR product prepared by overlap extension PCR was flanked by approximately 40 base pairs of target sequences containing the front part of the B5R gene and EGFP cassette. The GS1783-m8-BAC was electroporated with the PCR product to induce a Red recombination first and then cloned under the selective pressure of both 20 μg/ml of chloramphenicol and 25 μg/ml of kanamycin. A standard colony PCR was used to confirm whether the desired recombination successfully occurred. The release of the antibiotic-resistant gene from the co-integrate was achieved by a second Red recombination with the I-SceI digestion system, which was induced by the addition of 2% L-arabinose. The electrocompetent GS1783 retained each of pLC16m8.OS-BAC and pLC16m8.8S-BAC were used for the construction of pLC16m8.O-BAC and pLC16m8.8-BAC, respectively. A PCR product, which contained an antibiotic-resistant gene against zeocin with an I-SceI site flanked by approximately 40 base pairs of target sequences containing the rear part of the B5R gene and the untranslated region besides the B5R gene, were prepared using pUC-Zeo-S as the template. Subsequent Red recombination procedures were the same as described above except for using 25 μg/ml of zeocin instead of using kanamycin.

### Recovery of the infectious viruses from the BAC

First, 2.5 x 10^5^ 293FT cells were transfected with the 0.5 μg of the purified VAC-BAC plasmid with 1.5 μl of FuGENE 6 (Promega, Fitchburg, WI), and were then immediately infected with the fowlpox virus at an MOI of either 0.2 or 1.0. The infected cells along with DMEM-5FBS were collected when the EGFP-positive plaques were confirmed on the monolayer of the cells by fluorescent microscopy at 4 to 5 d.p.i. The cells were freeze-thawed 3 times to prepare a recovered virus stock.

### Confirmation of the release of EGFP and mini-F cassettes from the genome by conventional PCR

The viral genomic DNAs were collected from the RK13 cells infected with the recovered VAC at an MOI of 0.1 by the Hirt’s extraction protocol. The purified DNAs were added to PCR reaction mixtures of KOD Fx Neo (TOYOBO, Osaka, Japan), which contained a PCR buffer, 0.4 mM of dNTPs, 0.02 unit/μl of KOD FX Neo, H_2_O, and 250 mM of specific primer sets, A (5'-CCACCATGACACTAAGTTGC-3') with either B (5'-CCCTTGCTCACCATGAATTC-3') or C (5'-AAAATGCTCTAACGGCATCG-3'). After being denatured at 94°C for 2 min, 40 cycles of amplification were performed under the following conditions: denaturing at 94°C for 10 sec, annealing at 63°C for 30 sec, and extension at 68°C for 30 sec, followed by a 1°C decrease of the annealing temperature for the first five cycles and maintenance of the annealing temperature at 58°C for the rest of the cycles. After electrophoresis on 1% agarose gels, the PCR products with the expected sizes (737 base pairs by the primer sets A and B or 801 base pairs by primer sets A and C) were visualized by staining with GelRed. The sequence of the PCR products was confirmed using the Sanger sequencing technique with a 3500 Genetic Analyzer (Thermo Fisher Scientific).

### Full-genome sequencing of the VAC-BAC plasmids

The VAC-BAC plasmids were sequenced by a short-read sequencer, the Ion PGM system. The library from the VAC-BAC plasmids was prepared using the Ion Xpress Plus gDNA fragment library preparation chemistry. Briefly, a total of 0.1 or 1 μg of the purified plasmids was fragmented to a median size of 200 to 300 base pairs using Ion Shear Plus Reagents (Thermo Fisher Scientific). The fragmented DNAs were adapter-ligated and nick-repaired using the Ion Plus Fragment Library Kit with the Ion Xpress Barcode Adapters 1–16 Kit (Thermo Fisher Scientific). The libraries were size-selected with 2% of the E-Gel SizeSelect Agarose gel for a 200 base read (Thermo Fisher Scientific) and sequenced on an Ion PGM system using Ion 314 Chip v2 (Thermo Fisher Scientific). The sequencing data obtained were utilized to perform a reference-guided alignment with the m8 reference sequence from GenBank using the coverage Analysis (software version 5.0.2.0) and variant Caller (version 5.0.3.5) on a Torrent Server (Thermo Fisher Scientific) based on the default parameters. The mean of coverage depth of each sample was x 30 to x 170. The pLC16m8.8S-BAC was also sequenced on a long-read next-generation sequencer, PacBio RS II (Pacific Biosciences of California, Menlo Park, CA) by Macrogen Japan Corp. (Kyoto, Japan). Briefly, 5 μg of the purified pLC16m8.8S-BAC was fragmented to a median of 20-kilo base pairs by the Covaris g-TUBE device. Next, a 20-kilo base library was prepared from the purified DNA using the 20 kb SMRTbell Template Prep Kit 1.0 (Pacific Biosciences of California, Inc.) and sequenced on an RS II. The draft full-genome sequence was obtained by *de novo* assembly using HGAP (software version 2.2.0), which is available on either the DDBJ read annotation pipeline server or Canu (software version 1.3). The draft consensus sequences were then polished by a reference-guided alignment using RS_Resequencing program integrated into SMRT analysis software (software version 2.3.0) based on the default parameters. The annotations to the polished full-genome sequence were transferred from the VAC strain Copenhagen (accession no. M35027) using software, Genome Annotation Transfer Utility (GATU) [30].

### Reconfirmation of nucleotide sequence

After conventional PCR, three portions of the nucleotide sequence differences were observed between the m8 reference sequence and pLC16m8-BAC, and were confirmed by Sanger sequencing. Briefly, the viral genome DNA was extracted from 8.5 μl of a stock solution of m8 (1.0 x 10^8^ pfu/ml of viruses in DMEM-5 FBS) by proteinase K treatment. Conventional PCR using SapphaireAmp fast PCR master mix was performed with the following primer sets: for the nucleotide position 162321 (Forward; 5'-TGGATTTTTGATGGTGGTTTAACGT-3', reverse; 5'-TGGGCAGCGTTCACATTTTG-3'), for the position 6222 to 6243 (Forward; 5'-GCGCTTTCTCTATGGGTCCA-3', reverse; 5'-TGTCCACTTATGAGAAAACGTCA-3'), and for the position 182958 (Forward; 5'-CCCATGGGTAACACCAAGGT-3', reverse; 5'-TCTTCGGCGGTTTTCATGGA-3'). After the PCR products with the expected sizes (282 base pairs, 385 base pairs, 460 base pairs, respectively) were confirmed by agarose gel electrophoresis, and the nucleotide sequences were determined.

### Restriction fragment analysis

The purified BAC plasmid DNA was digested with *Nco*I and separated by electrophoresis for 2 hours at 50 V in 0.75% agarose/Tris-borate-EDTA buffer. A 1-kb DNA ladder (New England Biolabs) was used as molecular weight marker. Prediction of the restriction fragment patterns was computed by a software SnapGene version 4.0.6 (GSL Biotech).

### Full-genome sequencing of a viral clone from pLC16m8.8S-BAC

A plaque-purified clone of the GFP-negative vLC16m8.8S (8.8S-clone4), which was expanded two times (i.e., passage no. 2) in RK13 cells, was used for the viral genomic DNA preparation. A solution (1 ml) containing 2 x 10^8^ pfu of the virus was prepared from the infected cells by freeze-thawing the cells in the culture supernatant. After removal of the large debris by centrifugation, the virus solution was treated with the addition of 5 μl of Micrococcal nuclease (New England BioLabs) and 50 μl of 10 x buffer for 1 hour at 37°C to digest the genomic DNA originating from the infected RK13 cells. The viral genomic DNA was then purified from the solution using the High Pure Viral Nucleic Acid Kit (Roche Diagnostics, Indianapolis, IN), according to the manufacturer's protocol without the addition of the carrier RNA into the binding buffer. The purified DNA was amplified by the Illustra GenomiPhi V2 Kit (GE Healthcare, Chicago, IL), according to the manufacturer's protocol. The amplified DNA was electrophoresed in 0.75% of Seaplaque low melting agarose, and then a fragment of the gel, which contained the visualized high molecular weight DNA (more than 10-kilo base pairs), was excised and digested with thermostable beta-agarase (Nippon Gene co., ltd., Tokyo, Japan). The DNA in the digested agarose solution was purified by 2-propanol precipitation and then was fragmented to a median of 10-kilo base pairs by the Covaris g-TUBE device. The DNA was used to prepare a 10-kilo base library for next-generation sequencing using the 10 kb SMRTbell Template Prep Kit 1.0 (Pacific Biosciences of California, Inc.). The prepared templates were sequenced on a PacBio RS II by Macrogen Japan Corp. The sequencing data were utilized to perform a reference-guided alignment with the m8 reference sequence from GenBank using RS_Resequencing integrated into SMRT analysis software (software version 2.3.0) based on the default parameters.

### Confirmation of the ITR sequence structure of the authentic m8 and the recovered clone

The authentic m8 and the 8.8S-clone4 were each expanded once from the stock virus (passage no. 1). The virus solutions (20 ml each) were obtained from the infected cells by freeze-thawing the cells along with culture supernatant. After a low-speed centrifugation at 3,000 rpm to remove cell debris, the solutions were centrifuged at 10,000 rpm for 90 minutes. The pellets were suspended in solution I of the Sepagene DNA Extraction Kit (EIDIA, Tokyo, Japan), and the subsequent extraction procedures followed the protocol recommended by the manufacturer. Then, 1 μg of purified DNA was fragmented to a median of 300 to 500 base pairs using the BioRuptor (CosmoBio. Ltd., Tokyo, Japan). Libraries from the fragmented DNAs were prepared using the NEBNext Ultra DNA library Prep Kit for Illumina (NEB, New England Biolabs, Japan) with index primers from the NEBNext Multiplex Oligos for Illumina (NEB, Tokyo, Japan), according to the manufacturer’s instructions. Libraries were applied to a short-read next-generation sequencer, Illumina Miseq (Illumina, SanDiego, CA) by 250bp paired-end sequencing with the Miseq Reagent Kit v2 (500cycle). The data were mapped to the left ITR sequence extracted from pLC16m8-BAC as the reference sequence using the Burrows-Wheeler Aligner (0.7.15) with BWA-MEM algorithm [31] based on the default parameters.

### Statistical analysis

One-way analysis of variance (ANOVA) with Bonferroni's multiple-comparison test was used to test the equality of the means of the coverages of regions in the ITR using the GraphPad Prism 7 software program (GraphPad software, La Jolla, CA). A significant difference was considered to be present for any *P* value of <0.05. In addition, the magnitude of the difference between the groups (i.e., the effect size, r) was calculated based on Cohen’s description [32, 33]. The *r* value indicates < 0.1; very small, > = 0.1 to < 0.3; small, > = 0.3 to < 0.5; intermediate, or > 0.5; strong.

## Results

### Generation of a VAC-BAC plasmid, which harbors the full-length m8 genome

**Fig 1:**
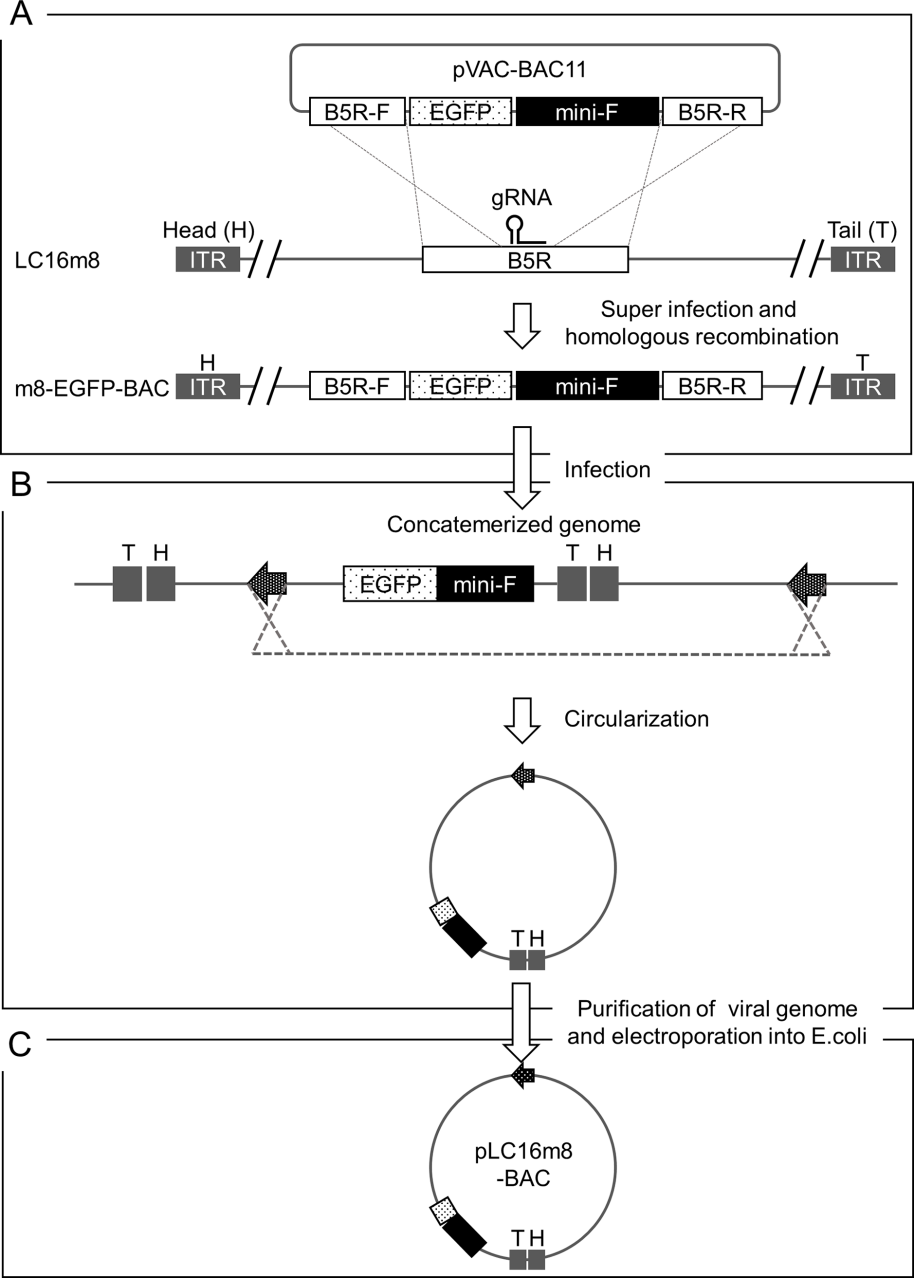
Scheme to generate a VAC-BAC plasmid, pLC16m8-BAC. A recombinant VAC, m8-EGFP-BAC, was generated by homologous recombination accelerated by a CRISPR-Cas9 system (A). pVAC-BAC11 harbors an EGFP expression cassette driven by an early and late VAC promoter and a mini-F cassette, which is essential for plasmid maintenance in *E*. *coli*. The cassettes were flanked by the front (B5R-F) and rear (B5R-R) part of the VAC B5R gene. Note that the gRNA targeted the sequence on the m8 genome but not on the pVAC-BAC11. m8-EGFP-BAC was used to infect RK13 cells (B). The virus genome formed replication intermediates, concatemers, some of which were circularized was purified from the infected RK13 cells with the addition of IβT. An electrocompetent *E*. *coli* was transformed by a circularized virus genome (arrows) of the concatemerized genome, and the chloramphenicol-resistant cells were cloned (C). The VAC-BAC plasmids obtained from the clones were named pLC16m8-BAC.

Homologous recombination was promoted by the CRISPR-Cas9 system for VAC to generate a recombinant m8, which harbors the EGFP and mini-F cassettes (m8-EGFP-BAC) (Fig 1A). 293FT cells were infected with plasmids, pVAC-BAC11, pVAC-T7pol-Cas9.1, and m8. The pVAC-BAC11 harbored the EGFP and mini-F cassettes and produced a gRNA, which targeted the specific sequence in the m8 B5R gene under the control of a T7 RNA polymerase promoter. The pVAC-T7pol-Cas9.1 produces Cas9 nuclease and T7 polymerase that were driven independently by a synthetic VAC early/late promoter. RK13 cells in monolayer culture were infected with crude virus stock for plaque purification of the clonal m8-EGFP-BAC. A total 17 clones were identified by EGFP expression under fluorescent microscopy and then purified. PCR confirmed the presence of the mini-F cassette. Then, 1 of the 17 clones was used for the following experiments. Extrachromosomal DNA was purified from the RK13 cells infected with m8-EGFP-BAC with the addition of IβT to inhibit viral hairpin resolution and accumulate unresolved concatemeric replication intermediates (Fig 1B). A total of 24 clones of chloramphenicol-resistant *E*. *coli* were obtained following electroporation of the purified DNA into electrocompetent cells (Fig 1C). The clones were, at least, guaranteed to harbor a mini-F cassette around the nucleotide position 160,000 in the plasmid genome since the mini-F cassette was indispensable for maintaining itself in *E*. *coli*. A conventional multiplex PCR was performed to confirm whether the VAC-BAC plasmid harbored the full-genome of m8, named pLC16m8-BAC. The PCR primer sets covered approximately from position 20,000 to 120,000 of the pLC16m8-BAC sequence in a fragmentary fashion (S Materials and methods and S1A Fig). As shown in S1B Fig, 3 out of 24 clones were PCR positive. Clones no. 3 and 4 seemed to harbor the entire region of the m8 genome, whereas clone, no. 20 lacked this genome at approximately position 20,000 to 40,000.

### Sequence comparison between the clones of pLC16m8-BAC and the authentic m8

The full-genome sequence of pLC16m8-BAC clones no. 3, 4, and 20 were determined by a short-read NGS. The nucleotide sequences of clones no. 3 and 4 were identical. There were two differences between clones no.3 and 4 and m8 reference sequence from GenBank, except for the EGFP and mini-F cassettes (Table 1). The EGFP and mini-F cassettes were confirmed to be inserted in the expected genomic location of these 3 clones. The sequence characteristics of clone no. 20 were identified as follows. The nucleotide position from 1 to 71,458 of the authentic m8 was absent. The position from 97,875–189,158 was duplicated, and a duplication was connected in a reverse orientation to the position 71,459. Next, the two sequence differences between the clones no.3 and 4, and the authentic m8 were re-confirmed by Sanger sequencing since the passaging history was different between the m8 used for generating pLC16m8-BAC and that used for determining the reference sequence deposited in the GenBank. The sequence at position 162,321 was confirmed to be A but not T in both authentic m8 and the clone of m8-EGFP-BAC used for making pLC16m8-BAC (S2 Fig). A single but not duplicate peak of the sequence chromatogram at position 162,321 suggested that a sequence variant did not exist in the m8 used in this study. Also, since the position from 6,222 to 6,243 was closely located on the left side of the inverted terminal repeat (ITR) of the genome, 23 plaque-purified clones from m8 were analyzed for the sequences around the position from 6,222 to 6,243 and the position 182,958, which might be the flip-flop part of the 6,222 to 6,243. Authentic m8 included three kinds of the sequence variants that consist of the combination of presence or absence of 15 base pair sequences (Table 2). Hence, it was confirmed that the pLC16m8-BAC clones no. 3 and no. 4 harbor the identical sequence of a variant of the authentic m8 genome, which did not contain a sequence (GGTAGACGAAGGTTAACCTGAT) in both ITRs.

**Table 1:** Sequence differences of VAC-BAC clones no. 3 and no. 4 from authentic LC16m8 except for the EGFP-BAC region.

Position	Sequence		Affected ORF
	Genbank reference	VAC-BAC clones	
6222–6243	GGTAGACGAAGGTTAACCTGAT	deleted	001R for m8
162321	T	A	A53R (S11T) for CPN, A54L (R21S) for CPN*, A ORF T (S11T) for CPN

*; Vaccinia virus Copenhagen strain (Genbank accession no. M35027)

**Table 2:** Sequence variants in the ITR regions from the stock of the authentic LC16m8.

		ITR right no. of clones (% of abundance)	
		Present*	Absent*
ITR left	Present	6 (26%)	15 (65%)
	Absent	0 (0%)	2 (9%)

*; The sequence (GGTAGACGAAGGTTAACCTGAT) is present or absent in the viral genome.

### Recovery of an infectious VAC from pLC16m8-BAC

**Fig 2:**
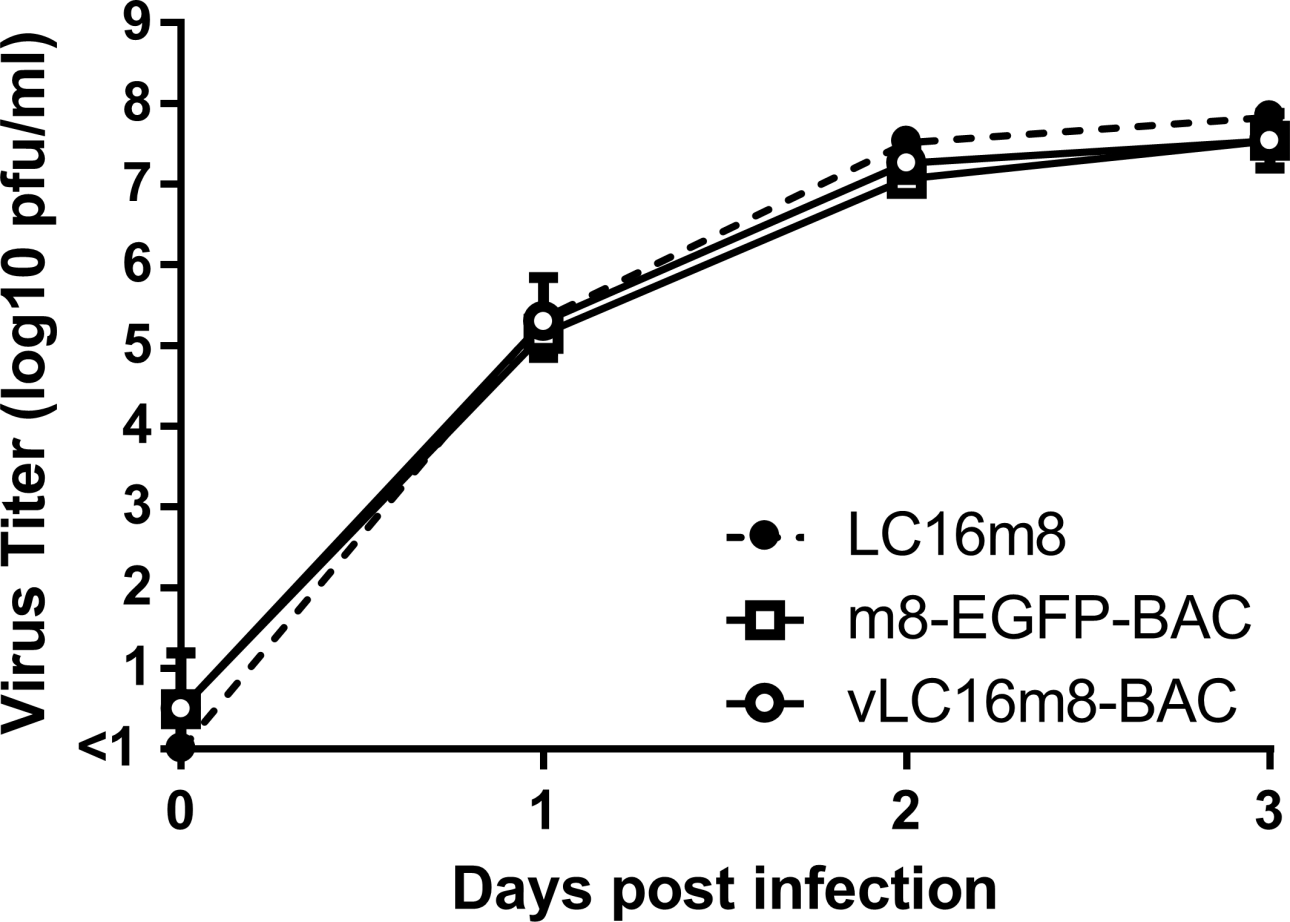
Growth of the recovered virus from pLC16m8-BAC named vLC16m8-BAC. RK13 cells were infected with either authentic m8, m8-EGFP-BAC, or vLC16m8-BAC at an MOI of 0.01. The infected cells along with the culture media at the indicated d.p.i. were collected and freeze-thawed. The amount of the virus was determined by a standard plaque assay.

For recovery of the infectious VAC from the clones of pLC16m8-BAC, fowlpox virus, which abortively infects mammalian cells, was used as the helper virus to supply transcriptional machinery. 293FT cells were infected with fowlpox virus, immediately after transfection of either pLC16m8-BAC clones no. 3, 4, 20 or the pVAC-BAC11 as a negative control. Four wells of the cells in the culture plate were prepared for each of the plasmid transfections to evaluate how many wells the infectious m8 could be recovered from. The apparent focuses or plaques formed with the accumulation of the EGFP-positive cells could be observed at four to 5 d.p.i., when the cells were transfected with clones no. 3 or 4 but not with clone no. 20 or pVAC-BAC11. The recovery of infectious virus in the freeze-thawed cells along with the culture media collected from each of the wells was confirmed by the standard plaque assay using RK13 cells (Table 3). The infectious virus recovery was 100%, when clones no. 3 and 4 were used, resulting in the recovery of infectious viruses at the dose of approximately 1 x 10^3^ pfu/ml in DMED-5FBS. In contrast, no infectious virus was recovered, when either clone no. 20, which did not harbor a large portion of the m8 genome, or the control pVAC-BAC11 were used. Next, the growth kinetic of the virus recovered from pLC16m8-BAC clone no. 3, named vLC16m8-BAC, was compared with that of the authentic m8. RK13 cells were infected with either m8, vLC16m8-BAC, or m8-EGFP-BAC at an MOI of 0.01. The infected cells along with DMED-5FBS were collected until 3 d.p.i., and the freeze-thawed cells with the culture media were subjected to the standard plaque assay to determine the viral growth curve (Fig 2). No apparent difference was observed between each of the growth curves. The infectious VAC, vLC16m8-BAC, could be recovered using pLC16m8-BAC clones no. 3 and 4, and the growth of the virus was indistinguishable from the authentic m8.

**Table 3:** Recovery of infectious viruses from the VAC-BAC clones.

	No. of wells with confirmed recovery / no. of wells used for the experiment	
	Experiment 1*	Experiment 2
Clone no. 3	4/4	4/4
Clone no. 4	4/4	4/4
Clone no. 20	0/4	Not done
pVAC-BAC11	0/4	Not done

*; Each experiment was performed independently.

### Generation of an m8-BAC system, which enables recovery of the bacterial replicon-free infectious virus

**Fig 3:**
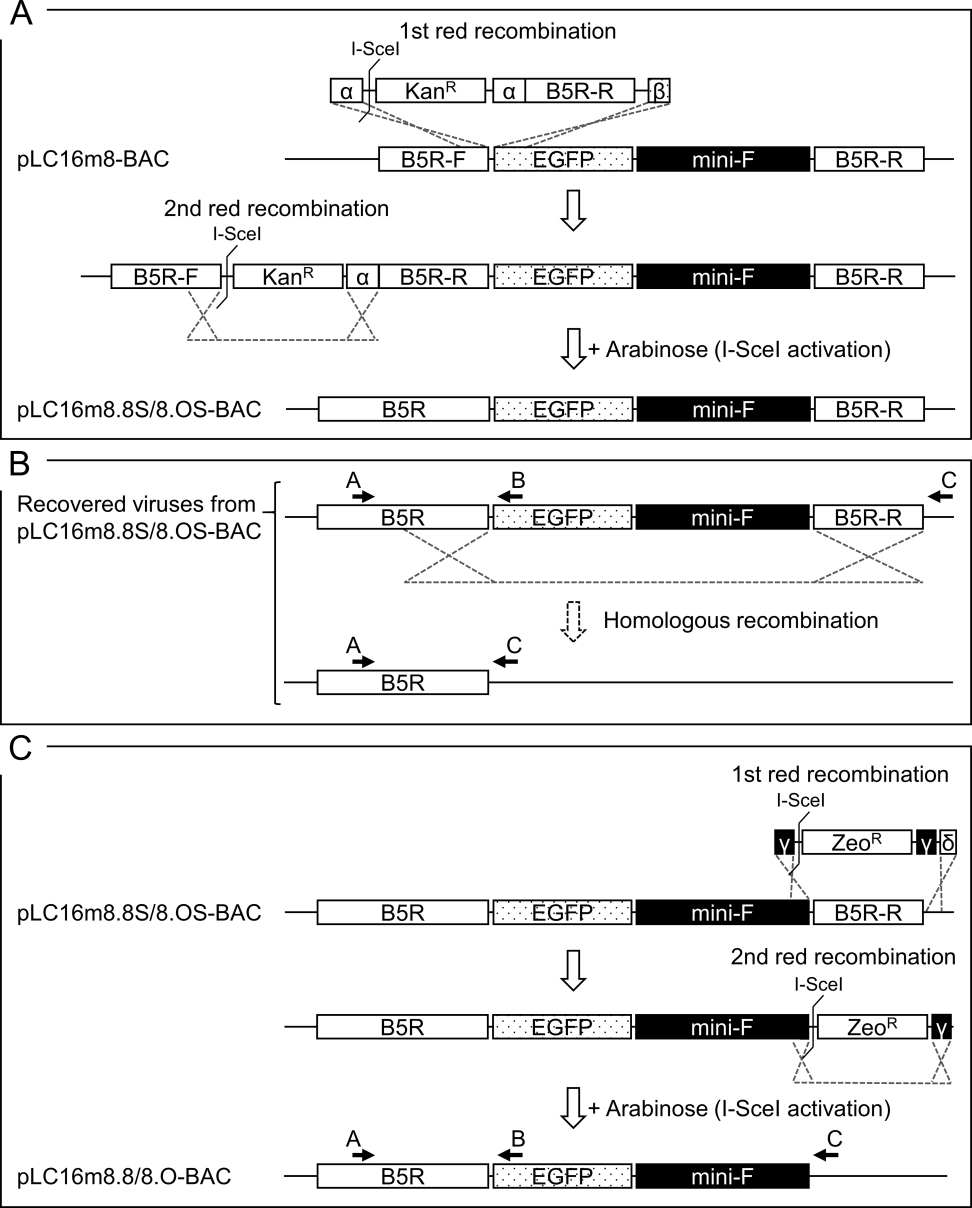
Generation of the m8-BAC system, which enables recovery of the bacterial replicon-free m8. The scheme to modify pLC16m8-BAC to pLC16m8.8S/8.OS-BAC and then to pLC16m8.8/8.O-BAC is shown. The first recombination was performed to insert PCR products of I-SceI site and a gene expression cassette conferred kanamycin resistance (Kan^R^) with the rear part of B5R (B5R-R) harbored by flanks homologous to parts of B5R-F and EGFP (α and β in the rectangles) cassette into pLC16m8-BAC (A). The flank α contained either of the nucleotide sequences that resulted defective (m8 type) or functional (mO type) B5R gene product. The second red recombination, which was achieved by induction of I-SceI digestion system with the addition of L-arabinose was performed to make both pLC16m8.8S-BAC and pLC16m8.OS-BAC by excision of the Kan^R^. Either m8 type or mO type B5R gene product was expressed, when the virus was recovered from either the pLC16m8.8S-BAC or pLC16m8.OS-BAC, and the EGFP and mini-F cassettes are expected to be self-excised by the homologous recombination while recovering the viruses (B). pLC16m8.8-BAC and pLC16m8.O-BAC were made from pLC16m8.8S-BAC and pLC16m8.OS-BAC, respectively (C). The first Recombination was performed to remove the B5R-R from either pLC16m8.8S-BAC or pLC16m8.OS-BAC by substitution of a PCR product. The PCR product harbored a gene expression cassette conferred zeocin resistance (Zeo^R^), which was flanked by homologous to part of B5R-R and the untranslated region besides B5R-R (γ and δ in the rectangles). Both pLC16m8.8-BAC and pLC16m8.O-BAC were made by excision of the Zeo^R^ by the second red recombination. The virus recovered from pLC16m8.8-BAC or pLC16m8.O-BAC is expected to stably harbor the EGFP and mini-F cassettes. The target region of primer A, B, and C used for following the PCR are indicated.

**Fig 4:**
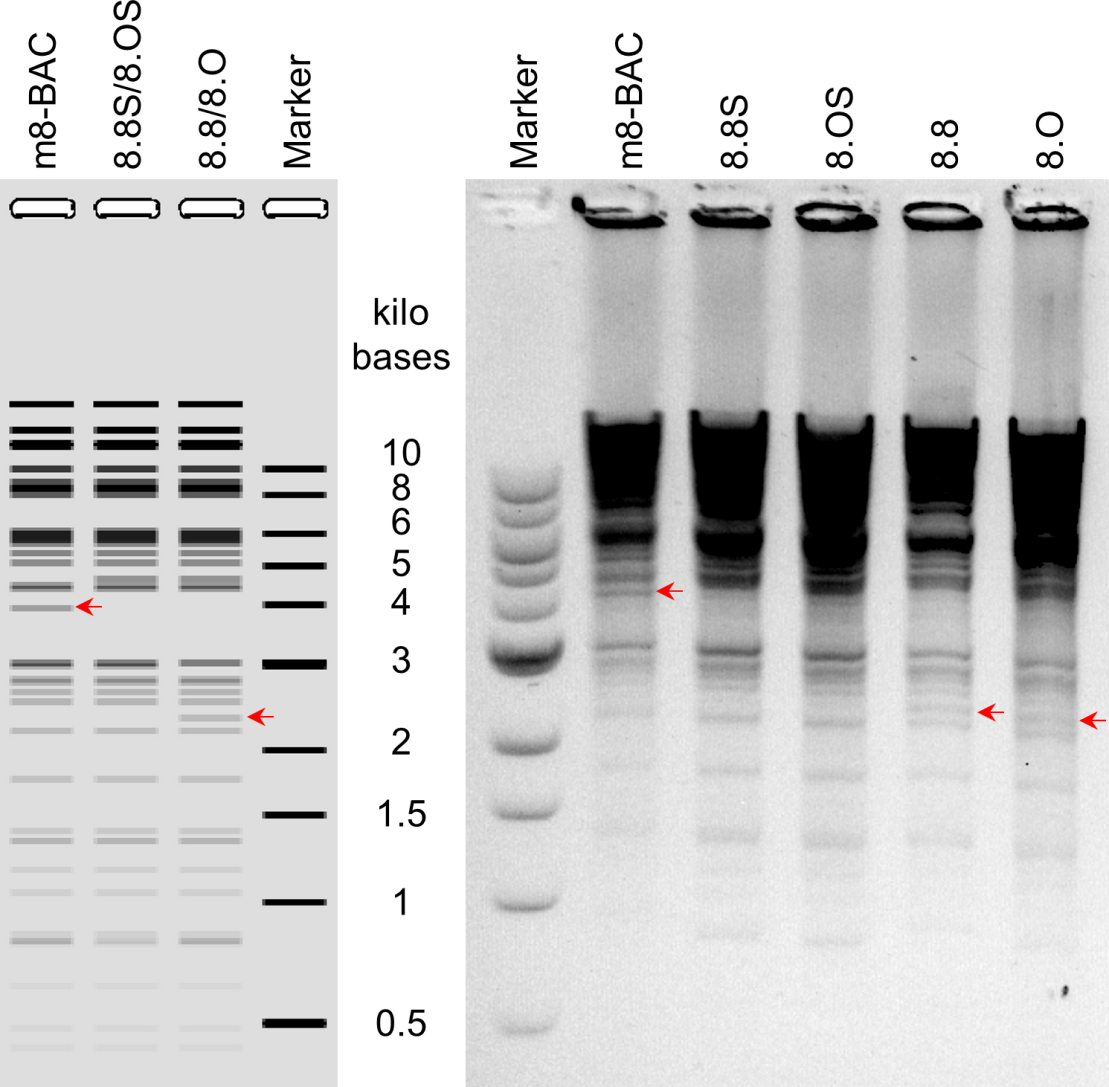
Restriction fragment analysis of BAC plasmids. Prediction of NcoI digested fragment patterns based on the sequences of pLC16m8-BAC and the derivatives were shown (A). Purified BAC plasmids, pLC16m8-BAC (m8-BAC), pLC16m8.8S/8.OS-BAC (8.8S or 8.OS) and pLC16m8.8/8.O (8.8/8.O) were digested with NcoI and separated on a 0.75% agarose gel (B). Color of the gel was inverted. Sizes of a molecular weight marker are shown as marker, and the sizes are given. Changes in the restriction pattern due to the presence of EGFP, mini-F cassettes or rear part of B5R are indicated with a red arrow.

**Fig 5:**
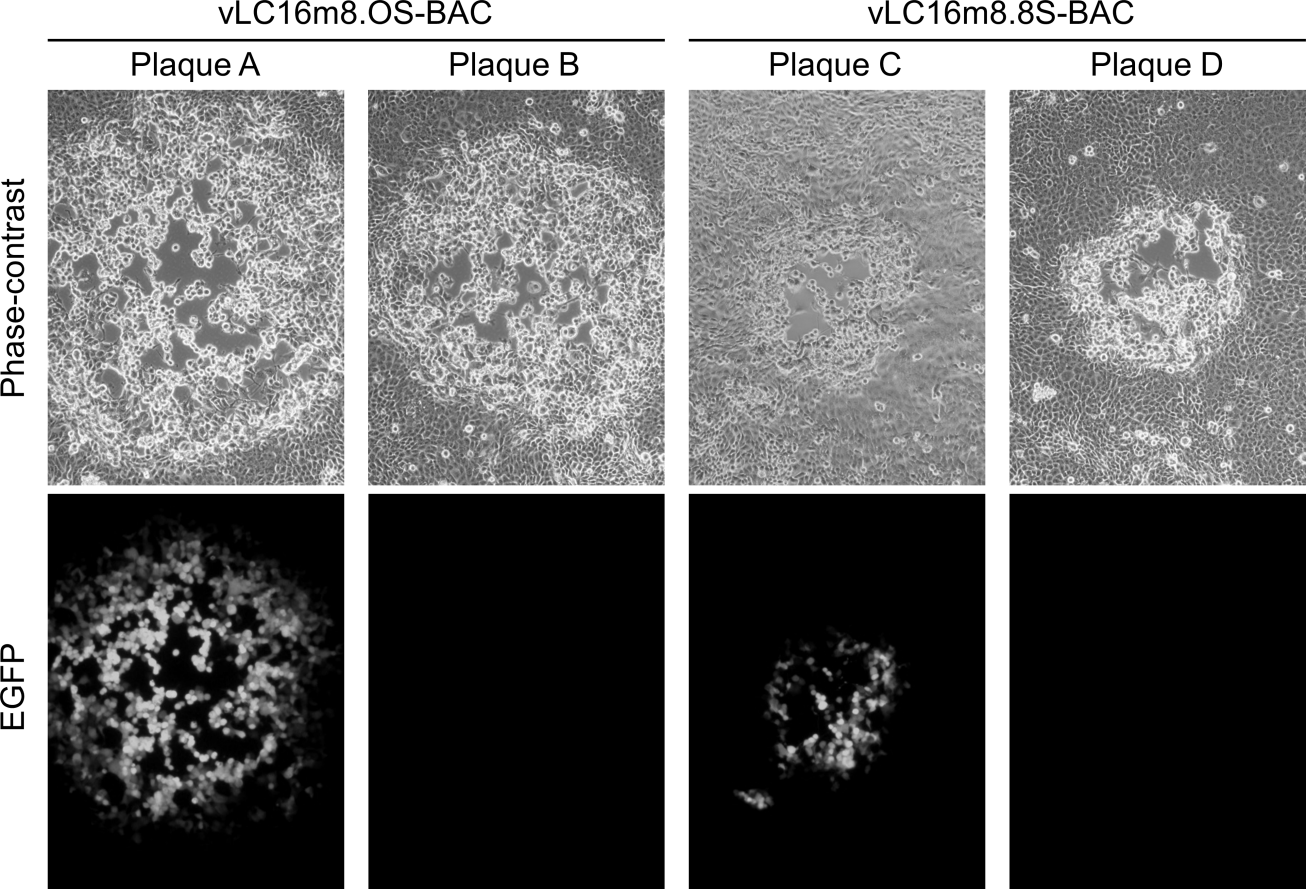
The characteristics of the viruses recovered from the pLC16m8.8S-BAC or pLC16m8.OS-BAC. The plaque size and EGFP expression were observed under phase-contrast and fluorescent microscopy. RK13 cells were infected with the recovered virus stocks from either pLC16m8.OS-BAC (vLC16m8.OS-BAC) or pLC16m8.8S-BAC (vLC16m8.8S-BAC). The plaque size and EGFP expression of vLC16m8.OS-BAC (plaque A and B) and vLC16m8.8S-BAC (plaque C and D) were observed at the same magnification under phase-contrast with fluorescent microscopy at 2 d.p.i.

**Fig 6:**
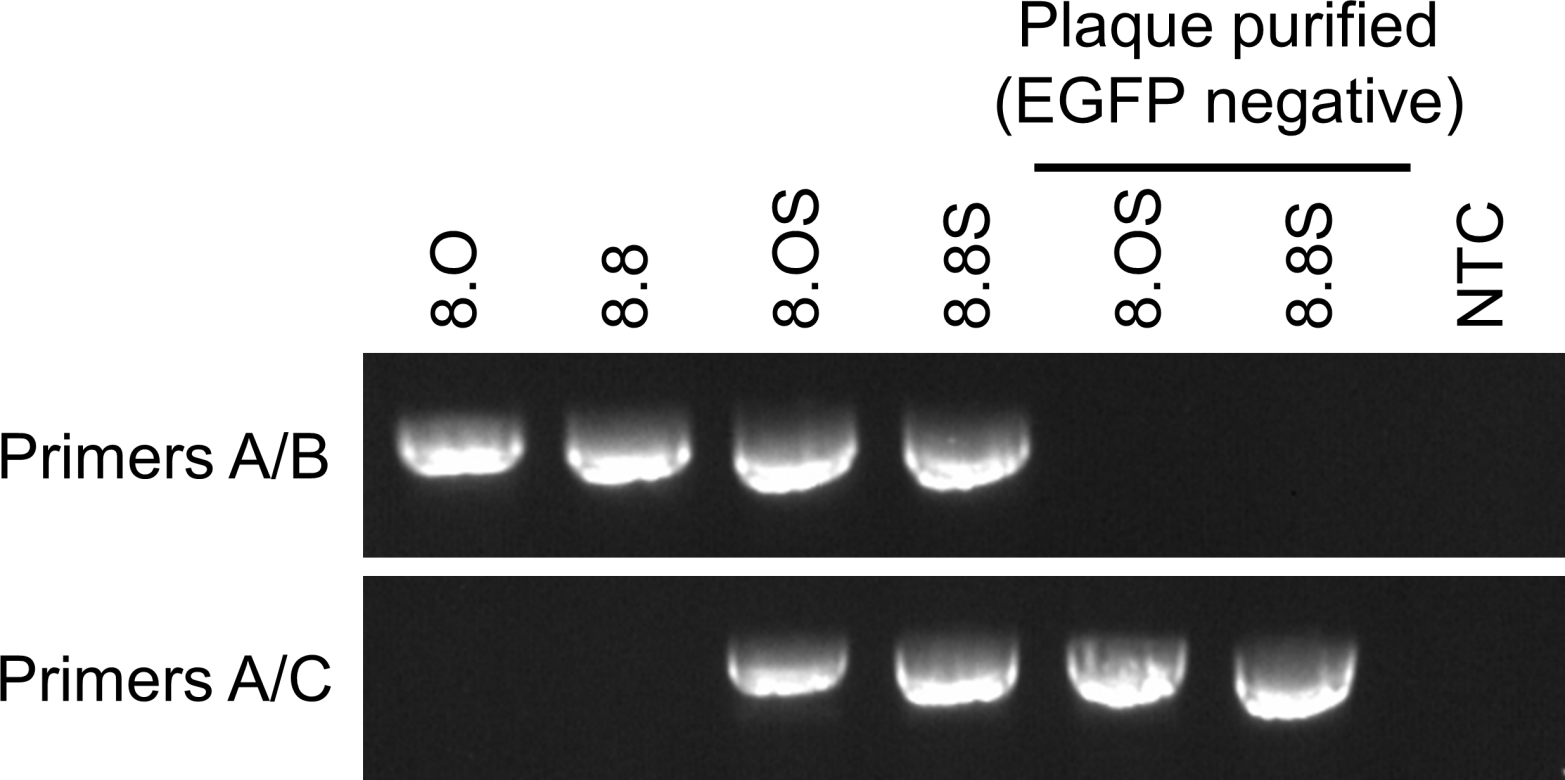
Confirmation of self-excising EGFP and mini-F cassettes from the virus genome. The viral genomic DNA purified from the RK13 cells infected with either vLC16m8.O-BAC (crude viruses) (8.O), vLC16m8.8-BAC (crude viruses) (8.8), vLC16m8.OS-BAC (crude or an EGFP-negative plaque cloned viruses) (8.OS), or vLC16m8.8S-BAC (crude or an EGFP-negative plaque cloned viruses) (8.8S) was amplified by conventional PCR using either the primer set A/B or A/C. The target position of the primers is shown in Fig 3A and 3B. The no template control (NTC) monitors unintentional PCR amplification to produce a false-positive result.

**Fig 7:**
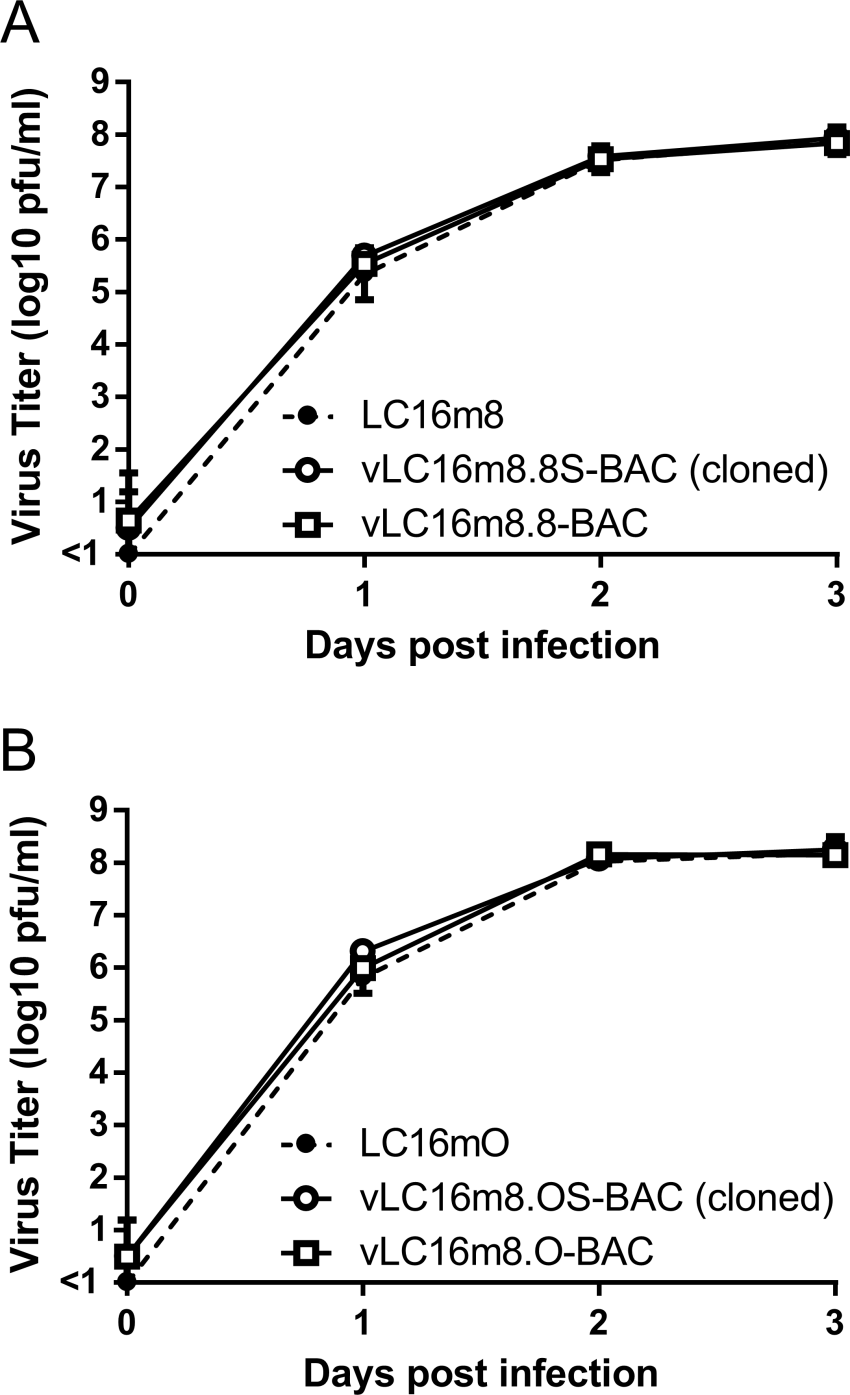
**Growth of the recovered viruses in comparison with that of authentic m8 (A) and mO (B).** RK13 cells were infected with either m8, vLC16m8.8-BAC (crude viruses), vLC16m8.8S-BAC (EGFP-negative plaque cloned), mO, vLC16m8.O-BAC (crude viruses), or vLC16m8.OS-BAC (EGFP-negative plaque cloned) as indicated at an MOI of 0.01.

A two-step markerless Red recombination system was adopted to modify pLC16m8-BAC to generate an advanced m8-BAC system, which enables recovery of the bacterial replicon-free m8 by spontaneous release of the EGFP and mini-F cassettes from the virus genome. In addition, as a characteristic of m8, a single-nucleotide deletion, which exists in the B5R envelope protein gene, results in a dysfunction of the protein. The dysfunction contributes to the attenuation of m8 in comparison with that of the LC16mO parental strain (mO) [5, 6, 9, 34]. Therefore, two kinds of plasmids that carry either a single-nucleotide deletion in B5R (pLC16m8.8S-BAC and pLC16m8.8-BAC) or the parental mO type B5R (pLC16m8.OS-BAC and pLC16m8.O-BAC) were generated. First, pLC16m8.8S-BAC and pLC16m8.OS-BAC were generated by insertion of the rear part of B5R (B5R-R) to the front part of B5R (B5R-F) to pLC16m8-BAC clone no. 3 (Fig 3A). Self-excision by homologous recombination to release the EGFP and mini-F cassettes in these VAC-BAC plasmids was not expected to occur or if so, the plasmid was expected to be diluted during collection of the plasmids in *E*. *coli* since the mini-F cassette was indispensable. On the other hand, self-excision was expected to occur in the recovered infectious virus genome (Fig 3B). Next, pLC16m8.8-BAC and pLC16m8.O-BAC were generated by removal of the B5R-R for stationary harboring the EGFP and mini-F cassettes in the recovered infectious virus genome (Fig 3C). Comparison of a restriction enzyme pattern obtained for the BAC plasmids and the recovered viruses confirmed to the presence of EGFP, mini-F cassettes or rear part of B5R (Fig 4). The pattern of *Nco*I digestion was matched those from the in silico predictions. Insertion of the rear part of B5R into pLC16m8-BAC to generate pLC16m8.8S/8.OS-BAC led to losing a 4.0-kilobase pairs band. Deletion of B5R-R from pLC16m8.8S/8.OS-BAC to generate pLC16m8.8/8.O-BAC led to an additional a 2.3-kilobase pairs band. Using next-generation sequencing, the 4 VAC-BAC plasmids were confirmed to be correctly generated without any undesired mutation. EGFP expression and the plaque size of the recovered viruses derived from each of the 4 plasmids were examined to confirm whether the system worked as expected. RK13 cells were infected with each of the recovered viruses, and the plaques on the cells were observed under phase-contrast with fluorescent microscopy at 2 d.p.i. The plaques formed by the recovered viruses from pLC16m8.8S-BAC (vLC16m8.8S-BAC) were smaller than those from pLC16m8.OS-BAC (vLC16m8.OS-BAC) as expected (Fig 5). A plaque size difference was also observed between the viruses from pLC16m8.8-BAC (vLC16m8.8-BAC) and those from pLC16m8.O-BAC (vLC16m8.O-BAC). Both EGFP-positive and -negative plaques were observed, when the cells were infected with either the crude vLC16m8.8S-BAC or vLC16m8.OS-BAC stock at passage number zero (i.e., the crude virus stock obtained from the infected 293FT cells) (Fig 5). The abundance of both the EGFP-negative vLC16m8.8S-BAC and vLC16m8.OS-BAC was approximately 5 to 10%. In contrast, only EGFP-positive plaques were observed, when the RK-13 cells were infected with vLC16m8.8-BAC or vLC16m8.O-BAC. The spontaneous release of the EGFP and mini-F cassettes from the recovered virus genome was evaluated by conventional PCR. After infection of RK13 cells with each virus at an MOI of 0.1, the viral genomic DNAs were purified from the RK13 cells. The PCR reactions were performed using either primer set A/B or A/C to distinguish whether the virus genome harbored the EGFP and mini-F cassettes or not (Fig 3B and 3C). The expected size of amplicons was confirmed from the template DNA, which was purified from the cells infected with either the crude vLC16m8.8/8.O-BAC or vLC16m8.8S/8.OS-BAC, with the primer set A/B (Fig 6). However, no amplification was confirmed from the DNA, which was purified from the cells infected with the clones from the EGFP-negative plaque of vLC16m8.8S/8.OS-BAC, with the primer set A/B. In addition, the amplicons were confirmed from the DNA, which was purified from the cells infected with both the crude and the plaque-purified vLC16m8.8S/8.OS-BAC, with the primer set A/C. Sanger sequencing of the PCR products also confirmed that the EGFP cassette and mini-F cassettes were properly self-excised. The growth of the recovered viruses was compared with that of authentic m8 and mO. RK13 cells were infected with either m8, mO, the crude vLC16m8.8/8.O-BAC, or the plaque-purified vLC16m8.8S/8.OS-BAC at an MOI of 0.01. The infected cells along with the culture media were collected until 3 d.p.i., and the freeze-thawed cells along with the culture media were subjected to the standard plaque assay (Fig 7). No apparent difference was observed in the growth kinetics among the viruses tested, m8, mO, the crude vLC16m8.8/8.O-BAC, and the plaque-purified vLC16m8.8S/8.OS-BAC. These results proved that the m8-BAC system, in which the bacterial replicon-free m8 could be recovered, was established.

### A long-read NGS of pLC16m8.8S-BAC

**Fig 8:**
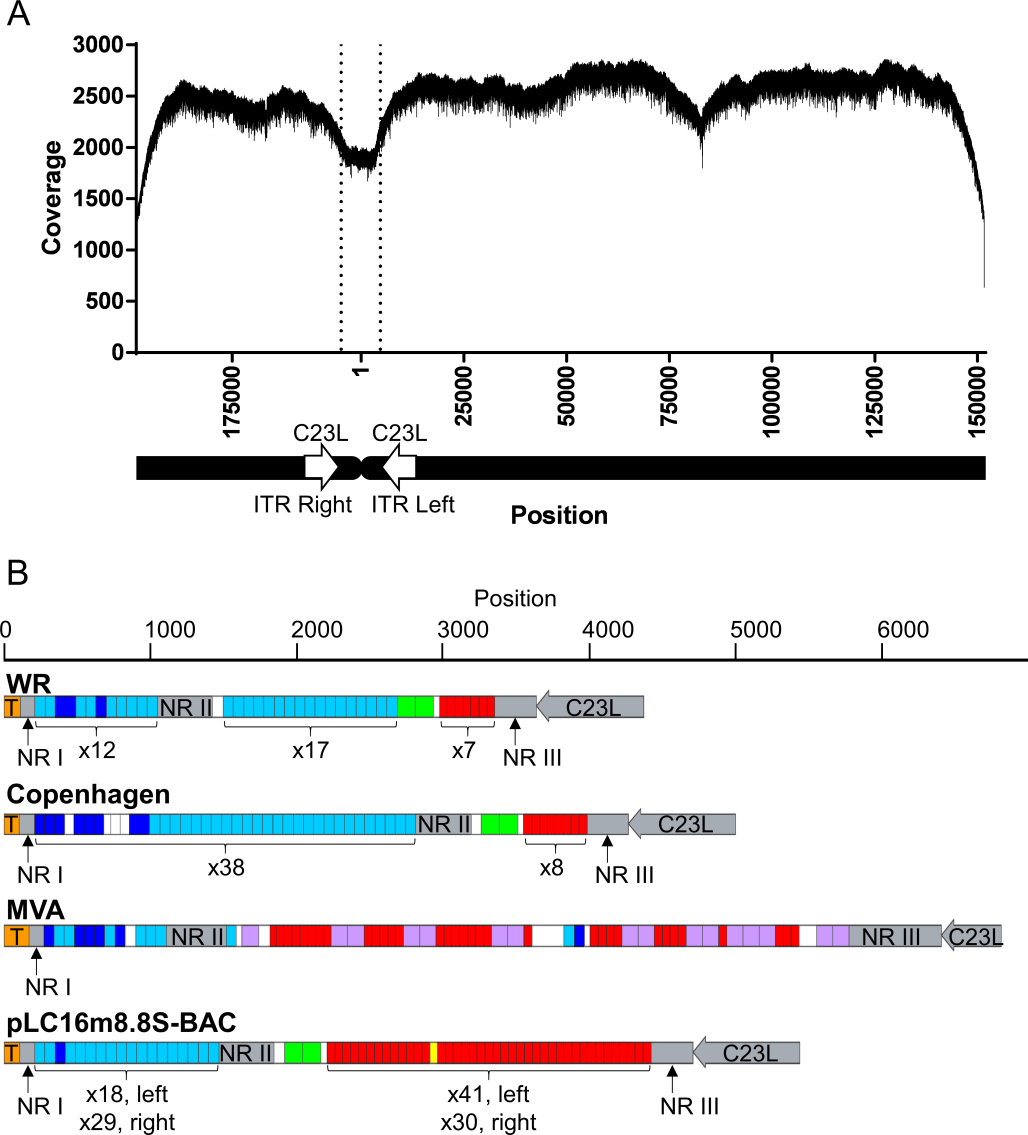
**Coverage depth of the pLC16m8.8S-BAC aligned to the consensus sequence of pLC16m8.8S-BAC generated by de novo assembly using HGAP software (A).** The ITR regions were not arranged at the termini of the sequence for the purpose of accurate reference-based alignment. The ITR left to the right region flanked by the C23L genes is indicated by vertical dotted lines. Nucleotide position 1 is at the terminus of the ITR left. The structure of the ITR left region to the C23L gene of pLC16m8.8S-BAC (accession no. LC315596), VAC strain WR (accession no. AY243312), Copenhagen (accession no. M35027), and MVA (accession no. U94848) are shown (B). Gray rectangles indicate unique sequences in the ITR: non-repeated region (NR) I, II, III, and the C23L gene. The T in the ocher rectangle indicates a terminal hairpin. Dark or light blue rectangles correspond to 69 or 70 base pair tandem repeats. Red and yellow rectangles correspond to 53 or 54 base pair tandem repeats. Green rectangles correspond to 125 base pair repeats. Purple rectangles correspond 111 or 112 base pair repeats. The number of repeats in the left or right ITR is indicated below the repeats.

**Fig 9:**
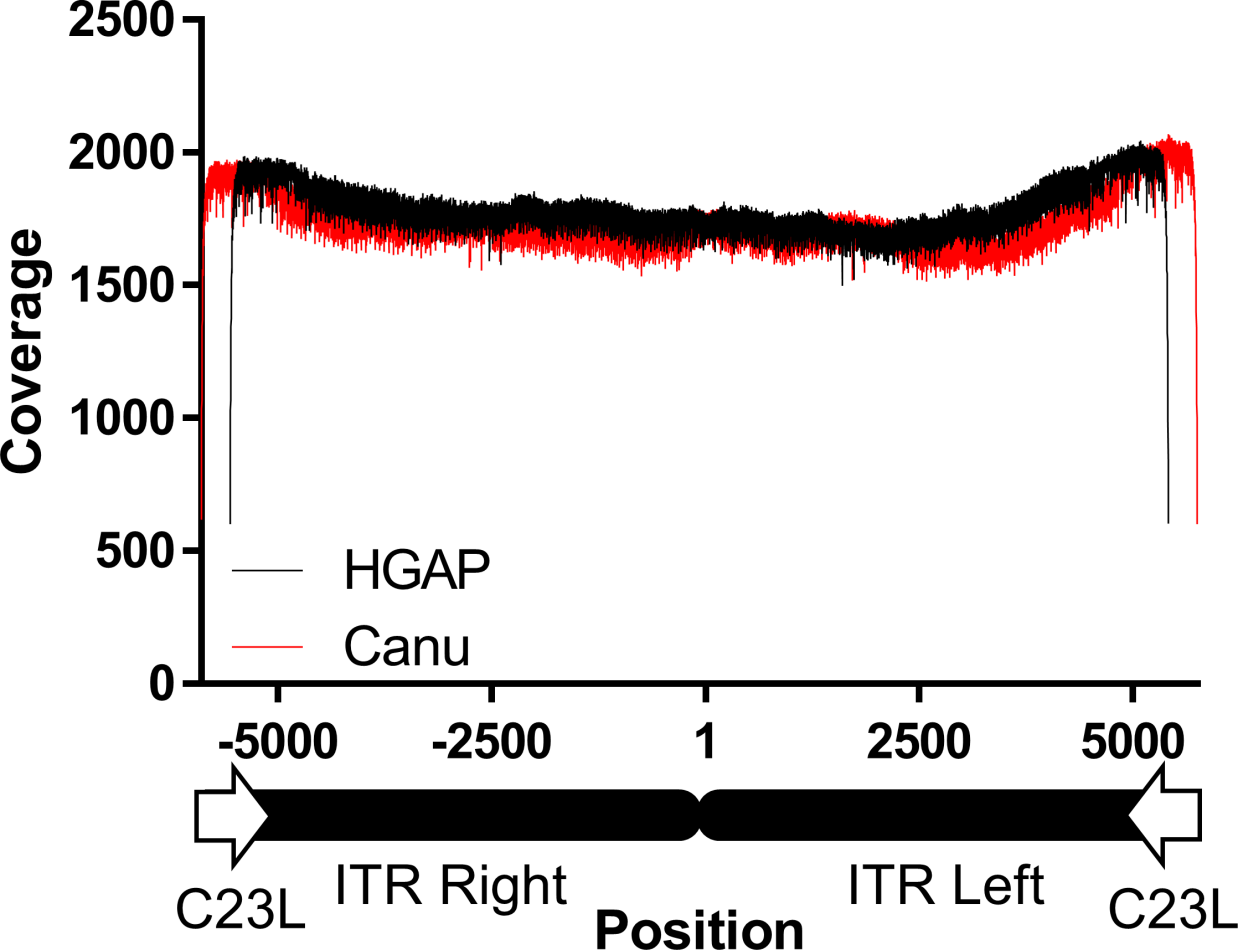
Coverage depth through the ITR left to the right region flanked by the C23L gene of pLC16m8.8S-BAC computed by HGAP (black line) or by Canu (red line).

A long-read NGS was performed to determine the entire sequence including the ITR of pLC16m8.8S-BAC. A contig covering the entire region of the plasmid was obtained from the raw reads by de novo assembly computed with software, HGAP or Canu that consist of different algorithms. The contigs were then polished to make the final consensus sequences by a reference-guided alignment, and the full-genome sequence of pLC16m8.8S-BAC was deposited at GenBank (accession no. LC315596). Although it was evident that the coverage depth was dropped at the left and right ITR region, the depth through the consensus sequence obtained from computation by HGAP was more than x 1,900 (Fig 8A). The distribution of coverage depth was similar to that aligned by Canu (S3 Fig). Both consensus sequences were identical except for the ITR region. The sequence of the hairpin terminus was identical to that of VAC WR strain. Also, the arrangement of the non-repeating regions, including I, II, III, 70 bp repeats, 125 bp repeats, and 54 bp repeats was relatively similar to those of the Copenhagen and WR strains rather than that of the MVA, which retains 111 or 112 base pair repeats (Fig 8B). The number of the repeats was different between the ITR left and right. Also, the number was diverse based on the information obtained by the software used for the de novo assembly, but the number of repeats was similar (Table 4). Hence, to validate the number of repeats, the ITR right and left regions spanned by C23L were extracted from the consensus sequences and applied for a reference-guided alignment again (Fig 9). Remarkable dropping or jumping of coverage at a site generally occur if the number of repeats in the reference is different from those obtained by the software. However, neither the coverage on the ITR regions computed by HGAP nor that computed by Canu showed any remarkable alteration.

**Table 4:** Number of 54 bp or 70 bp repeats in the ITR left or right.

De novo assembly software	Repeat number			
	69 or 70 bp		54 bp	
	ITR left	ITR right	ITR left	ITR right
HGAP	18	29	41	30
Canu	20	32	45	30

### Sequence identity between the authentic m8 and the recovered vLC16m8.8S-BAC

**Fig 10:**
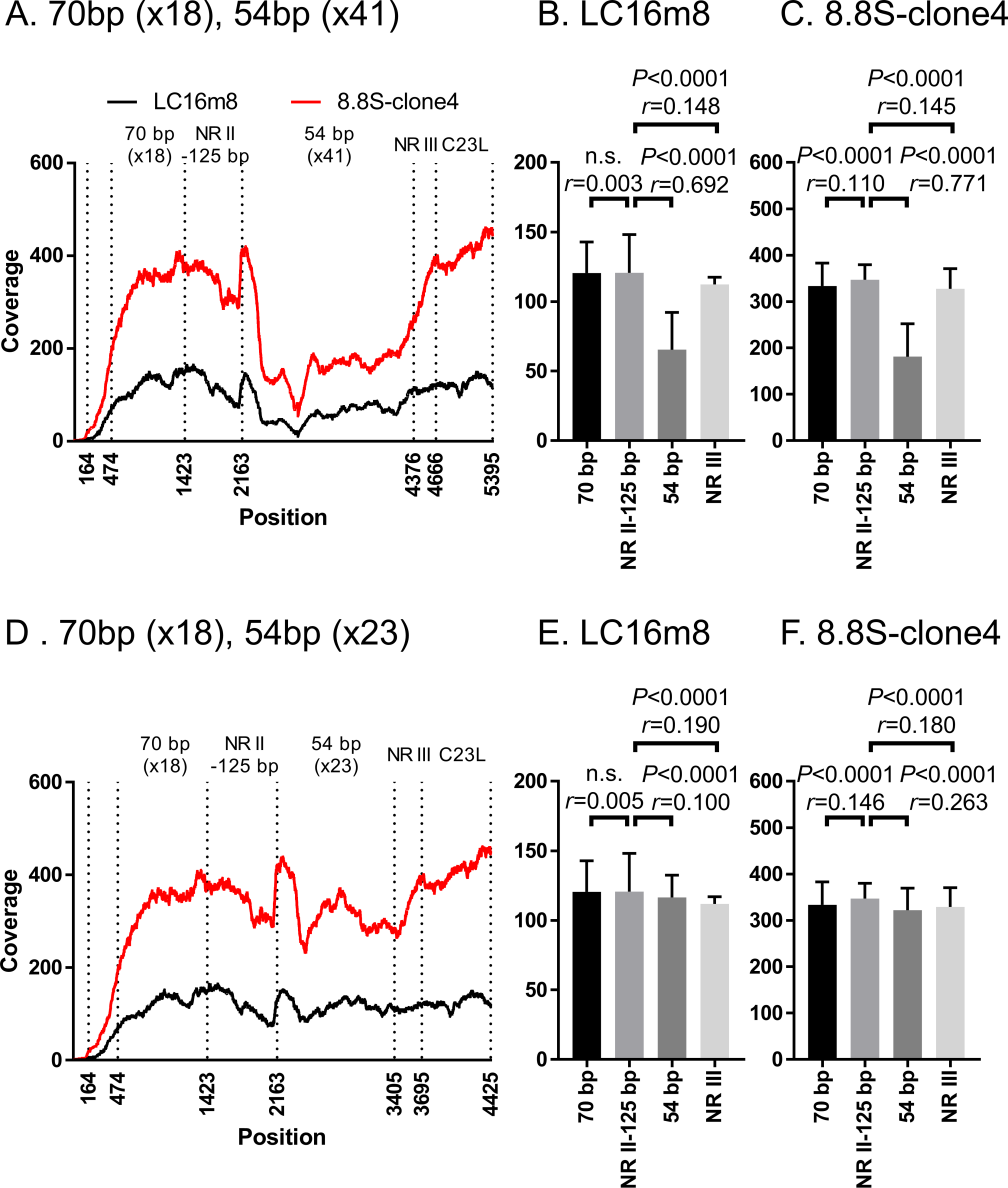
Identity of the ITR region in authentic m8 and 8.8S-clone4, a clone that was plaque-purified from EGFP-negative and bacterial replicon-free vLC16m8.8S-BAC. The coverage depth computed by the alignment of short-reads to the sequence of the left ITR region, which was extracted from the consensus sequence of pLC16m8.8S-BAC is shown (A and D). The coverage depth in the ITR left region from the terminus (position 1) to the C23L gene of m8 or 8.8S-clone 4 was plotted as either a black or a red line. The mean of the coverage depth by each of the regions (i.e., 70 base pair repeat, non-repeated region II to 125 base pairs, 54 base pair repeat, non-repeated region III, and C23L) was plotted (B, C, E, and F). One-way ANOVA with Bonferroni's multiple-comparison test was used to determine the level of statistical significance. The effect size (r), which indicates the magnitude of the mean difference between the regions, was also calculated. The mean of coverage depth at the 70 base pairs repeats were calculated from position 474 to 1423 to exclude the effect of low coverage depth artificially occurred at the terminus of the reference sequence. The number of 54 base pair tandem repeats in the reference sequence was artificially modified from x 41 (A-C) to x 23 (D-E). “n.s.” indicates "not significant”.

A long-read NGS was performed to confirm the sequence of a clone of vLC16m8.8S-BAC (8.8S-clone4), which was found to be EGFP-negative and bacterial replicon-free. For preparation of the sequencing sample, we used an original method, which could prepare a large amount of highly pure whole VAC genome from a small amount of virus-infected cell culture media by utilizing a micrococcal nuclease and phi29 DNA polymerase. The viral culture medium was treated with micrococcal nuclease, which was a non-specific endo-exonuclease to eliminate the host genome from the virus-infected cell culture media, but the viral genome was protected from the nuclease by the viral envelope. The purified DNA from the virus solution was then amplified by phi29 DNA polymerase, which has strand displacement and processive synthesis activity under isothermal conditions. After the NGS, a reference-guided alignment was performed using the m8 reference sequence. The average of the coverage depth aligned through the reference sequence was x 2,315 (S4 Fig). It was confirmed that the genome in the 8.8S-clone4 did not retain the EGFP and mini-F cassettes, and the rest of the region was identical to that of the m8 except for two portions as shown in Table 1. Next, after a 2 x 250 base paired library preparation from the concentrated viruses by centrifugation, a short-read NGS was performed to confirm the identity of the ITR sequence structure between the population of authentic m8 and that of the 8.8S-clone4. A reference-guided alignment was performed using the left ITR sequence extracted from the pLC16m8.8S-BAC, which was expected to be similar to that of the authentic m8. There was no remarkable difference observed in the pattern of the coverages aligned to the left ITR sequence between the authentic m8 and the 8.8S-clone4 (Fig 10A). Also, the averages of the coverage depth by each of the regions (i.e., 70 base pairs repeats, non-repeated region II to 125 base pairs, 54 base pairs repeat, non-repeated region III, and C23L as shown in Fig 8B) were calculated (Fig 10B and 10C). Although the equality of the means of the coverages of regions in the ITR was significantly different between most of the regions (i.e., *P* < 0.05), all of the effect sizes (i.e., *r* values), except for the comparison between the NR II-125 bp and 54 bp regions were less than 0.3. The small *r* values indicated that the magnitudes of the mean differences between the regions were small. This pattern similarity did not change even if the number of 54 base pairs tandem repeats in the reference sequence used for the alignment was artificially modified from x 41 to x 23 (Fig 10D). Also, the *r* value between NR II-125 bp and 54 bp regions became less than 0.3 (Fig 10E and 10F). These results proved that the plaque-purified clone from the EGFP-negative and bacterial replicon-free virus recovered from pLC16m8.8S-BAC was genotypically indistinguishable from the authentic m8.

## Discussion

The m8 and MVA are categorized into third generation smallpox vaccines that represent highly attenuated VAC strains developed as safer vaccines than those of the first and second generation smallpox vaccines [15]. In addition, m8 was recommended with ACAM2000, a derivative of NYCBH as the preferred WHO stockpile smallpox vaccine by the WHO Strategic Advisory Group of Experts (SAGE) meeting on immunization in 2013 [35]. These facts encouraged us to elucidate the attenuation mechanism and to study its potential as a recombinant vaccine vector.

Self-excisable BAC system has already been described based on herpes- and poxviruses. Most of the systems utilize the Cre-loxP recombination system to accomplish self-excising the mini-F cassette [36–41]. However, one disadvantage is that the Cre-loxP system fundamentally requires 34 bases of the loxP sequence, which inevitably remains in the virus genome after the mini-F cassette excision. Hence, our VAC-BAC system was adopted from the self-excisable BAC systems by utilizing intramolecular homologous recombination [19, 25, 41]. A point of difference between the m8-BAC system and other systems is the complexity needed to prevent the loss of mini-F cassette in *E*. *coli*. The BAC plasmids in the previous studies were prevented from losing the mini-F cassettes in *E*. *coli* through the recombination by the inverse orientation of the internal sequence duplication with two antiparallel recombination events and the need for both the chloramphenicol resistance gene and the mini-F origin, *OriS* [19, 25, 41]. On the other hand, the mini-F cassette is simply flanked by the tandem of the sequence duplication in pLC16m8.8S-BAC and pLC16m8.OS-BAC, but the m8-BAC plasmids were stably maintained in *E*. *coli* under chloramphenicol pressure. Despite the stability of pLC16m8.8S-BAC and pLC16m8.OS-BAC in *E*. *coli*, the bacterial replicon-free infectious m8 was easily observed as EGFP-negative plaques even when the virus stock from the infected 293FT cells (i.e., passage no. 0) was used. Also, only one plaque cloning was enough to obtain a clone of bacterial replicon-free m8. This stability feature is a characteristic of the system for generating the recombinant m8 easily and quickly. Meanwhile, the growth of m8-EGFP-BAC, vLC16m8-BAC, vLC16m8.8-BAC, and vLC16m8.O-BAC did not differ from either the authentic m8 or mO. Hence, the property of EGFP expression is useful for some applications that are necessary for tracing the viral infection via the fluorescent signal.

The long-read NGS was easier to determine the ITR sequence of VAC than the other procedures performed previously [42–45]. However, determining the number of repeats in the ITR was still complicated. In spite of coverage depth of a plasmid, such as pLC16m8.8S-BAC, that should be uniform through the entire sequence, the depth was dropped at the ITR region (Fig 8). The reason is likely not because of the difficulty of library preparation, polymerase sequencing, or reference mapping, but it is likely that the 20 kilobase library preparation was not of sufficient length to determine the precise number of the repeats in the ITR. This speculation was supported by the distribution of mapped reads and the length of the ITR regions that were not remarkably different from that to the middle of the m8 genome (S5 Fig).

The number of 54 base pairs and 70 base pairs repeats between the ITR left and right of pLC16m8.8S-BAC was not equal. This observation supports the previous reports that described heterogeneity in the length of the VAC terminal fragments confirmed by agarose gel electrophoresis following restriction enzyme digestion [43, 46].

In general, accurately resolving tandem repeats by short-read NGS is a challenging task in genome alignment since the reads are too short to span the tandem repeats. A solution for this difficulty is to assume the genome to be uniformly covered (i.e., if the coverage depth through the viral genome is x 100, that of a tandem repeat must be x 100) [47]. In fact, the magnitudes of the mean coverage differences through the ITR region in both m8 and 8.8S-clone4 were small except for the 54 bp region, and the statistical significance was achieved because the sample number of the groups was large (i.e., every region contained more than > 740 bases). Also, the coverage depth of 54 base pairs tandem repeats seems to be uniform to the other region, when the number is x 23 rather than x 41 (Fig 10D–10F).

There are only six nucleotide differences between m8 and mO [5]. Three out of six were in noncoding regions, probably in promoter regions. One out of three nonsynonymous substitutions was a single-nucleotide deletion in the B5R. The B5R is a determinant of both neurovirulence in suckling mice and the plaque size in comparison of m8 with mO [5, 9, 34]. The other one out of three nonsynonymous substitutions was located at the nucleotide position 162,321 and involves an A (mO) to T (m8) substitution, which probably be a variation of the m8 sequence due to the difference in the passaging history (Table 1). The previous reports suggested that the phenotypical difference between m8 and mO largely depends on the B5R [5, 9, 34]. Interestingly, it is known that mO, like m8, already possesses the highly attenuated phenotype of neurovirulence for both rabbit and monkey and has higher temperature sensitivity in comparison with the grandparental strain, Lister [4, 6]. Therefore, the VAC-BAC system using pLC16m8.O-BAC and pLC16m8.OS-BAC that carries functional B5R is suitable for studying the attenuation mechanism, except for that of the B5R, which should be addressed.

In summary, a VAC-BAC system, in which the infectious m8 can be recovered was established. Four m8-BAC plasmids are currently available and depend on the functional B5R and/or the bacterial replicon-free or the EGFP expression. In particular, the bacterial replicon-free system using pLC16m8.8S-BAC is suitable for developing a recombinant vaccine vector, since unexpected side effects that may occur from the remaining unnecessary elements will be avoided. The entire genomic sequence of the recovered infectious virus was confirmed to be indistinguishable from that of authentic m8.

## Supporting information

S1 Materials and methods (DOCX)

S1 Fig Confirmation that the VAC-BAC plasmid clones harbored the full-genome of m8-EGFP-BAC. A schematic view of the target of multiplex PCR primer sets on m8-EGFP-BAC is shown (A). The size of the PCR products and the position of the target genes are indicated. The PCR products were visualized by electrophoresis (B). All four PCR products were confirmed from clones no. 3 and 4. Two of these were confirmed from clone no. 20.
(TIF)

S2 Fig Confirmation of the nucleotide sequence of authentic m8 and m8-EGFP-BAC. The chromatograms of m8 and m8-EGFP-BAC around position 162321 are shown.
(TIF)

S3 Fig Coverage depth of the pLC16m8.8S-BAC aligned to the consensus sequence of pLC16m8.8S-BAC generated by de novo assembly using Canu software. (TIF)

S4 Fig Coverage depth of 8.8S-clone4 aligned to the reference sequence of m8 from GenBank. (TIF)

S5 Fig Distribution of mapped read length at the ITR left to the right region. The region is flanked by C23L genes (A) and the middle of the m8 genome at position 80000 to 92000 (B).
(TIF)

## References

[1] Aragon TJ, Ulrich S, Fernyak S, Rutherford GW. Risks of serious complications and death from smallpox vaccination: a systematic review of the United States experience, 1963–1968. BMC Public Health. 2003;3:26 doi: 10.1186/1471-2458-3-26 ; PubMed Central PMCID: PMCPMC194634. 12911836 10.1186/1471-2458-3-26 PMC194634

[2] Yamaguchi M, Kimura M, Hirayama M. Report of the National Smallpox Vaccination Research Committee: study of side effects, complications and their treatment. Clinical Virology. 1975;3:269–78.

[3] Morita M, Aoyama Y, Arita M, Amona H, Yoshizawa H, Hashizume S, et al Comparative studies of several vaccinia virus strains by intrathalamic inoculation into cynomolgus monkeys. Arch Virol. 1977;53(3):197–208. . 404993 10.1007/BF01314664

[4] Kenner J, Cameron F, Empig C, Jobes DV, Gurwith M. LC16m8: an attenuated smallpox vaccine. Vaccine. 2006;24(47–48):7009–22. doi: 10.1016/j.vaccine.2006.03.087 . 17052815 10.1016/j.vaccine.2006.03.087 PMC7115618

[5] Morikawa S, Sakiyama T, Hasegawa H, Saijo M, Maeda A, Kurane I, et al An attenuated LC16m8 smallpox vaccine: analysis of full-genome sequence and induction of immune protection. J Virol. 2005;79(18):11873–91. doi: 10.1128/JVI.79.18.11873-11891.2005 ; PubMed Central PMCID: PMCPMC1212643. 16140764 10.1128/JVI.79.18.11873-11891.2005 PMC1212643

[6] Sugimoto M, Yasuda A, Miki K, Morita M, Suzuki K, Uchida N, et al Gene structures of low-neurovirulent vaccinia virus LC16m0, LC16m8, and their Lister original (LO) strains. Microbiology and immunology. 1985;29(5):421–8. . 2993828 10.1111/j.1348-0421.1985.tb00843.x

[7] Empig C, Kenner JR, Perret-Gentil M, Youree BE, Bell E, Chen A, et al Highly attenuated smallpox vaccine protects rabbits and mice against pathogenic orthopoxvirus challenge. Vaccine. 2006;24(17):3686–94. doi: 10.1016/j.vaccine.2005.03.029 . 16430997 10.1016/j.vaccine.2005.03.029

[8] Saijo M, Ami Y, Suzaki Y, Nagata N, Iwata N, Hasegawa H, et al LC16m8, a highly attenuated vaccinia virus vaccine lacking expression of the membrane protein B5R, protects monkeys from monkeypox. J Virol. 2006;80(11):5179–88. doi: 10.1128/JVI.02642-05 ; PubMed Central PMCID: PMCPMC1472157. 16698998 10.1128/JVI.02642-05 PMC1472157

[9] Yokote H, Shinmura Y, Kanehara T, Maruno S, Kuranaga M, Matsui H, et al Safety of attenuated smallpox vaccine LC16m8 in immunodeficient mice. Clin Vaccine Immunol. 2014;21(9):1261–6. doi: 10.1128/CVI.00199-14 ; PubMed Central PMCID: PMCPMC4178579. 24990910 10.1128/CVI.00199-14 PMC4178579

[10] Belyakov IM, Earl P, Dzutsev A, Kuznetsov VA, Lemon M, Wyatt LS, et al Shared modes of protection against poxvirus infection by attenuated and conventional smallpox vaccine viruses. Proc Natl Acad Sci U S A. 2003;100(16):9458–63. doi: 10.1073/pnas.1233578100 ; PubMed Central PMCID: PMCPMC170940. 12869693 10.1073/pnas.1233578100 PMC170940

[11] Earl PL, Americo JL, Wyatt LS, Eller LA, Whitbeck JC, Cohen GH, et al Immunogenicity of a highly attenuated MVA smallpox vaccine and protection against monkeypox. Nature. 2004;428(6979):182–5. doi: 10.1038/nature02331 . 15014500 10.1038/nature02331

[12] Wyatt LS, Earl PL, Eller LA, Moss B. Highly attenuated smallpox vaccine protects mice with and without immune deficiencies against pathogenic vaccinia virus challenge. Proc Natl Acad Sci U S A. 2004;101(13):4590–5. doi: 10.1073/pnas.0401165101 ; PubMed Central PMCID: PMCPMC384791. 15070762 10.1073/pnas.0401165101 PMC384791

[13] Gilbert SC. Clinical development of Modified Vaccinia virus Ankara vaccines. Vaccine. 2013;31(39):4241–6. doi: 10.1016/j.vaccine.2013.03.020 . 23523410 10.1016/j.vaccine.2013.03.020

[14] Stanley DA, Honko AN, Asiedu C, Trefry JC, Lau-Kilby AW, Johnson JC, et al Chimpanzee adenovirus vaccine generates acute and durable protective immunity against ebolavirus challenge. Nat Med. 2014;20(10):1126–9. doi: 10.1038/nm.3702 . 25194571 10.1038/nm.3702

[15] Walsh SR, Dolin R. Vaccinia viruses: vaccines against smallpox and vectors against infectious diseases and tumors. Expert Rev Vaccines. 2011;10(8):1221–40. doi: 10.1586/erv.11.79 ; PubMed Central PMCID: PMCPMC3223417. 21854314 10.1586/erv.11.79 PMC3223417

[16] Domi A, Moss B. Cloning the vaccinia virus genome as a bacterial artificial chromosome in Escherichia coli and recovery of infectious virus in mammalian cells. Proc Natl Acad Sci U S A. 2002;99(19):12415–20. doi: 10.1073/pnas.192420599 ; PubMed Central PMCID: PMCPMC129459. 12196634 10.1073/pnas.192420599 PMC129459

[17] Domi A, Moss B. Engineering of a vaccinia virus bacterial artificial chromosome in Escherichia coli by bacteriophage lambda-based recombination. Nat Methods. 2005;2(2):95–7. doi: 10.1038/nmeth734 . 15782205 10.1038/nmeth734

[18] Cottingham MG, Andersen RF, Spencer AJ, Saurya S, Furze J, Hill AV, et al Recombination-mediated genetic engineering of a bacterial artificial chromosome clone of modified vaccinia virus Ankara (MVA). PLoS One. 2008;3(2):e1638 doi: 10.1371/journal.pone.0001638 ; PubMed Central PMCID: PMCPMC2242847. 18286194 10.1371/journal.pone.0001638 PMC2242847

[19] Cottingham MG, Gilbert SC. Rapid generation of markerless recombinant MVA vaccines by en passant recombineering of a self-excising bacterial artificial chromosome. Journal of virological methods. 2010;168(1–2):233–6. doi: 10.1016/j.jviromet.2010.04.012 . 20417665 10.1016/j.jviromet.2010.04.012

[20] Moss B. Poxvirus DNA replication. Cold Spring Harb Perspect Biol. 2013;5(9). doi: 10.1101/cshperspect.a010199 ; PubMed Central PMCID: PMCPMC3753712. 23838441 10.1101/cshperspect.a010199 PMC3753712

[21] Chakrabarti S, Sisler JR, Moss B. Compact, synthetic, vaccinia virus early/late promoter for protein expression. BioTechniques. 1997;23(6):1094–7. . 9421642 10.2144/97236st07

[22] Tischer BK, von Einem J, Kaufer B, Osterrieder N. Two-step red-mediated recombination for versatile high-efficiency markerless DNA manipulation in Escherichia coli. BioTechniques. 2006;40(2):191–7. . 16526409 10.2144/000112096

[23] Tischer BK, Smith GA, Osterrieder N. En passant mutagenesis: a two step markerless red recombination system. Methods Mol Biol. 2010;634:421–30. doi: 10.1007/978-1-60761-652-8_30 . 20677001 10.1007/978-1-60761-652-8_30

[24] Yuan M, Zhang W, Wang J, Al Yaghchi C, Ahmed J, Chard L, et al Efficiently editing the vaccinia virus genome by using the CRISPR-Cas9 system. J Virol. 2015;89(9):5176–9. doi: 10.1128/JVI.00339-15 ; PubMed Central PMCID: PMCPMC4403460. 25741005 10.1128/JVI.00339-15 PMC4403460

[25] Roth SJ, Hoper D, Beer M, Feineis S, Tischer BK, Osterrieder N. Recovery of infectious virus from full-length cowpox virus (CPXV) DNA cloned as a bacterial artificial chromosome (BAC). Vet Res. 2011;42:3 doi: 10.1186/1297-9716-42-3 ; PubMed Central PMCID: PMCPMC3031225. 21314965 10.1186/1297-9716-42-3 PMC3031225

[26] Cottingham MG. Genetic manipulation of poxviruses using bacterial artificial chromosome recombineering. Methods Mol Biol. 2012;890:37–57. doi: 10.1007/978-1-61779-876-4_3 . 22688760 10.1007/978-1-61779-876-4_3

[27] Borst EM, Crnkovic-Mertens I, Messerle M. Cloning of beta-herpesvirus genomes as bacterial artificial chromosomes. Methods Mol Biol. 2004;256:221–39. doi: 10.1385/1-59259-753-X:221 . 15024169 10.1385/1-59259-753-X:221

[28] Hirt B. Selective extraction of polyoma DNA from infected mouse cell cultures. J Mol Biol. 1967;26(2):365–9. . 4291934 10.1016/0022-2836(67)90307-5

[29] Horton RM, Hunt HD, Ho SN, Pullen JK, Pease LR. Engineering hybrid genes without the use of restriction enzymes: gene splicing by overlap extension. Gene. 1989;77(1):61–8. . 2744488 10.1016/0378-1119(89)90359-4

[30] Tcherepanov V, Ehlers A, Upton C. Genome Annotation Transfer Utility (GATU): rapid annotation of viral genomes using a closely related reference genome. BMC Genomics. 2006;7:150 doi: 10.1186/1471-2164-7-150 ; PubMed Central PMCID: PMCPMC1534038. 16772042 10.1186/1471-2164-7-150 PMC1534038

[31] Li H. Aligning sequence reads, clone sequences and assembly contigs with BWA-MEM. Quantitative Biology. 2013:arXiv:1303.3997v2 [q-bio.GN].

[32] Cohen J. A power primer. Psychol Bull. 1992;112(1):155–9. . 19565683 10.1037//0033-2909.112.1.155

[33] Cohen J. Statistical Power Analysis for the Behavioral Sciences. 2nd ed. Hillsdale, NJ: Erlbaum; 1988.

[34] Kidokoro M, Tashiro M, Shida H. Genetically stable and fully effective smallpox vaccine strain constructed from highly attenuated vaccinia LC16m8. Proc Natl Acad Sci U S A. 2005;102(11):4152–7. doi: 10.1073/pnas.0406671102 ; PubMed Central PMCID: PMC554788. 15753319 10.1073/pnas.0406671102 PMC554788

[35] No-authors-listed. Meeting of the Strategic Advisory Group of Experts on immunization, November 2013—conclusions and recommendations. Wkly Epidemiol Rec. 2014;89(1):1–20. . 24466571

[36] Richards AL, Sollars PJ, Smith GA. New tools to convert bacterial artificial chromosomes to a self-excising design and their application to a herpes simplex virus type 1 infectious clone. BMC Biotechnol. 2016;16(1):64 doi: 10.1186/s12896-016-0295-4 ; PubMed Central PMCID: PMCPMC5006514. 27580861 10.1186/s12896-016-0295-4 PMC5006514

[37] Smith GA, Enquist LW. A self-recombining bacterial artificial chromosome and its application for analysis of herpesvirus pathogenesis. Proc Natl Acad Sci U S A. 2000;97(9):4873–8. doi: 10.1073/pnas.080502497 ; PubMed Central PMCID: PMCPMC18325. 10781094 10.1073/pnas.080502497 PMC18325

[38] Stanton RJ, Baluchova K, Dargan DJ, Cunningham C, Sheehy O, Seirafian S, et al Reconstruction of the complete human cytomegalovirus genome in a BAC reveals RL13 to be a potent inhibitor of replication. J Clin Invest. 2010;120(9):3191–208. doi: 10.1172/JCI42955 ; PubMed Central PMCID: PMCPMC2929729. 20679731 10.1172/JCI42955 PMC2929729

[39] Nagel CH, Dohner K, Fathollahy M, Strive T, Borst EM, Messerle M, et al Nuclear egress and envelopment of herpes simplex virus capsids analyzed with dual-color fluorescence HSV1(17+). J Virol. 2008;82(6):3109–24. doi: 10.1128/JVI.02124-07 ; PubMed Central PMCID: PMCPMC2258981. 18160444 10.1128/JVI.02124-07 PMC2258981

[40] Yu D, Smith GA, Enquist LW, Shenk T. Construction of a self-excisable bacterial artificial chromosome containing the human cytomegalovirus genome and mutagenesis of the diploid TRL/IRL13 gene. J Virol. 2002;76(5):2316–28. ; PubMed Central PMCID: PMCPMC153828. 11836410 10.1128/jvi.76.5.2316-2328.2002 PMC153828

[41] Tischer BK, Kaufer BB, Sommer M, Wussow F, Arvin AM, Osterrieder N. A self-excisable infectious bacterial artificial chromosome clone of varicella-zoster virus allows analysis of the essential tegument protein encoded by ORF9. J Virol. 2007;81(23):13200–8. doi: 10.1128/JVI.01148-07 ; PubMed Central PMCID: PMCPMC2169085. 17913822 10.1128/JVI.01148-07 PMC2169085

[42] Antoine G, Scheiflinger F, Dorner F, Falkner FG. The complete genomic sequence of the modified vaccinia Ankara strain: comparison with other orthopoxviruses. Virology. 1998;244(2):365–96. doi: 10.1006/viro.1998.9123 . 9601507 10.1006/viro.1998.9123

[43] Goebel SJ, Johnson GP, Perkus ME, Davis SW, Winslow JP, Paoletti E. The complete DNA sequence of vaccinia virus. Virology. 1990;179(1):247–66, 517–63. . 2219722 10.1016/0042-6822(90)90294-2

[44] Baroudy BM, Moss B. Sequence homologies of diverse length tandem repetitions near ends of vaccinia virus genome suggest unequal crossing over. Nucleic Acids Res. 1982;10(18):5673–9. ; PubMed Central PMCID: PMCPMC320915. 6292846 10.1093/nar/10.18.5673 PMC320915

[45] Baroudy BM, Venkatesan S, Moss B. Incompletely base-paired flip-flop terminal loops link the two DNA strands of the vaccinia virus genome into one uninterrupted polynucleotide chain. Cell. 1982;28(2):315–24. . 7060133 10.1016/0092-8674(82)90349-x

[46] Moss B, Winters E, Cooper N. Instability and reiteration of DNA sequences within the vaccinia virus genome. Proc Natl Acad Sci U S A. 1981;78(3):1614–8. ; PubMed Central PMCID: PMCPMC319182. 6262819 10.1073/pnas.78.3.1614 PMC319182

[47] Treangen TJ, Salzberg SL. Repetitive DNA and next-generation sequencing: computational challenges and solutions. Nat Rev Genet. 2011;13(1):36–46. doi: 10.1038/nrg3117 ; PubMed Central PMCID: PMCPMC3324860. 22124482 10.1038/nrg3117 PMC3324860

