# Supplementary material for: Construction and characterization of bacterial artificial chromosomes harboring the full-length genome of a highly attenuated vaccinia virus LC16m8
Source: PLoS One. 2018 Feb 23;13(2):e0192725. doi: 10.1371/journal.pone.0192725 (PMC5825015; doi:10.1371/journal.pone.0192725)
Supplement: S1 Materials and methods — (DOCX) [file pone.0192725.s001.docx]

S Materials and methods

Conventional multiplex PCR to confirm that the plasmid harbored the m8 genome

The 1 μl of purified plasmids was added to 9 μl of SapphireAmp Fast PCR master mix containing 1 x master mix, H_2_O, 100 nM of G9R, and 4 pairs of specific primer sets, (Forward; 5'-CAGACGTTCACGACACTGGA-3', reverse; 5'-AAGGCTGAACCGATCCACTG-3'), 120 nM of C3L (Forward; 5'-CCCGCTAGACAAGTATCCGT-3', reverse; 5'-GGAATCCCGAGGCACCTATT-3'), 150 nM of F5L (Forward; 5'-GTGGTGGTGTAGTAGAACCA-3', reverse; 5'-TATTTGTTGGCTGTATGCGG-3') and 200 nM of A4L (Forward; 5'-CCAGTAGGTGTAGGAGTACA-3', reverse; 5'-TAACAAGTTCTCACAGAGGC-3'). After electrophoresis on 1 % agarose gels, the PCR products with the expected sizes (G9R; 103 bp, C3L; 251 bp, F5L; 415 bp, A4L; 600 bp) were visualized by staining with GelRed.
