# Supplementary figures and images for: Construction and characterization of bacterial artificial chromosomes harboring the full-length genome of a highly attenuated vaccinia virus LC16m8
Source: PLoS One. 2018 Feb 23;13(2):e0192725. doi: 10.1371/journal.pone.0192725 (PMC5825015; doi:10.1371/journal.pone.0192725)

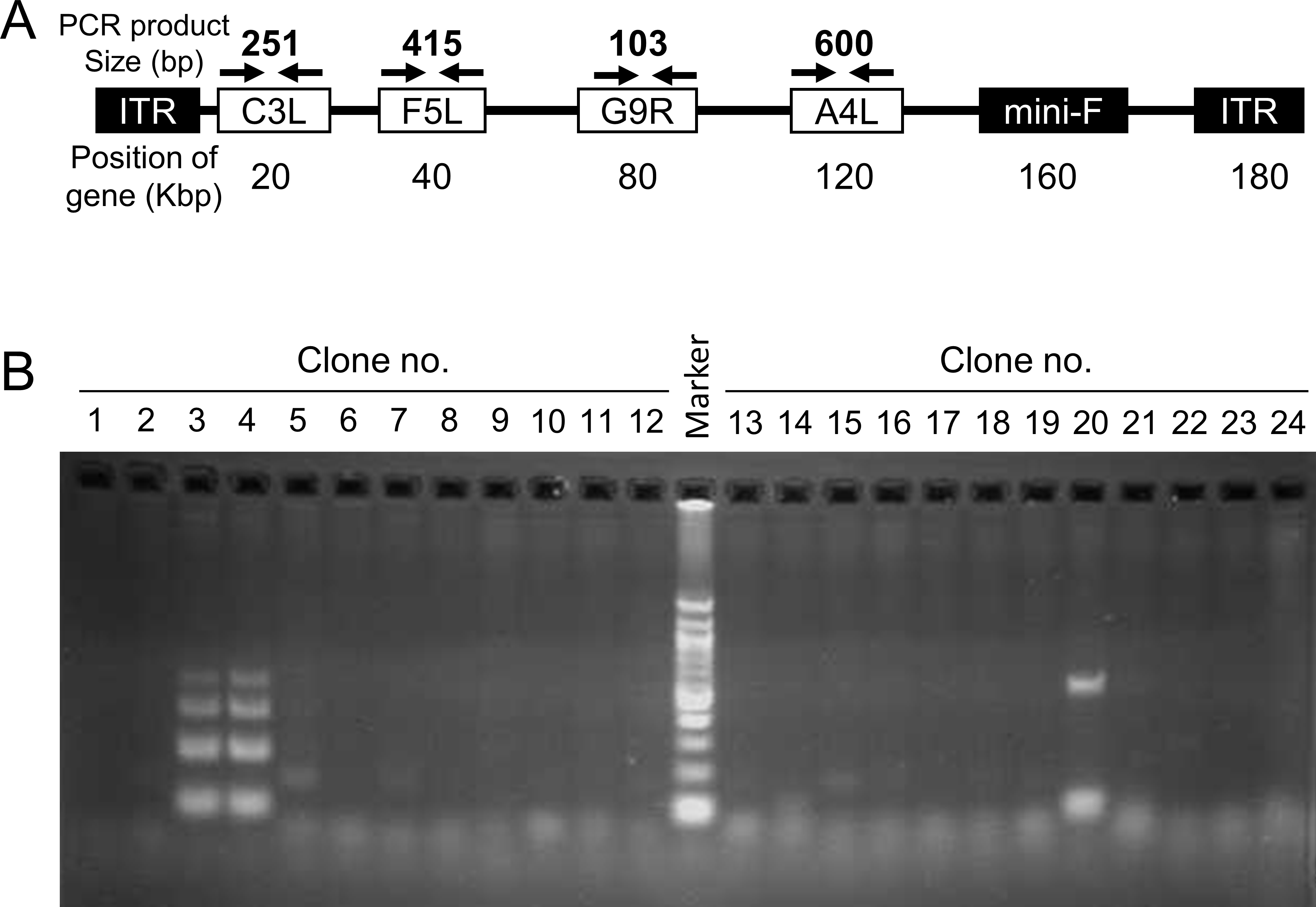

Supplement: S1 Fig — A schematic view of the target of multiplex PCR primer sets on m8-EGFP-BAC is shown (A). The size of the PCR products and the position of the target genes are indicated. The PCR products were visualized by electrophoresis (B). All four PCR products were confirmed from clones no. 3 and 4. Two of these were confirmed from clone no. 20. (TIF) [file pone.0192725.s002.tif]

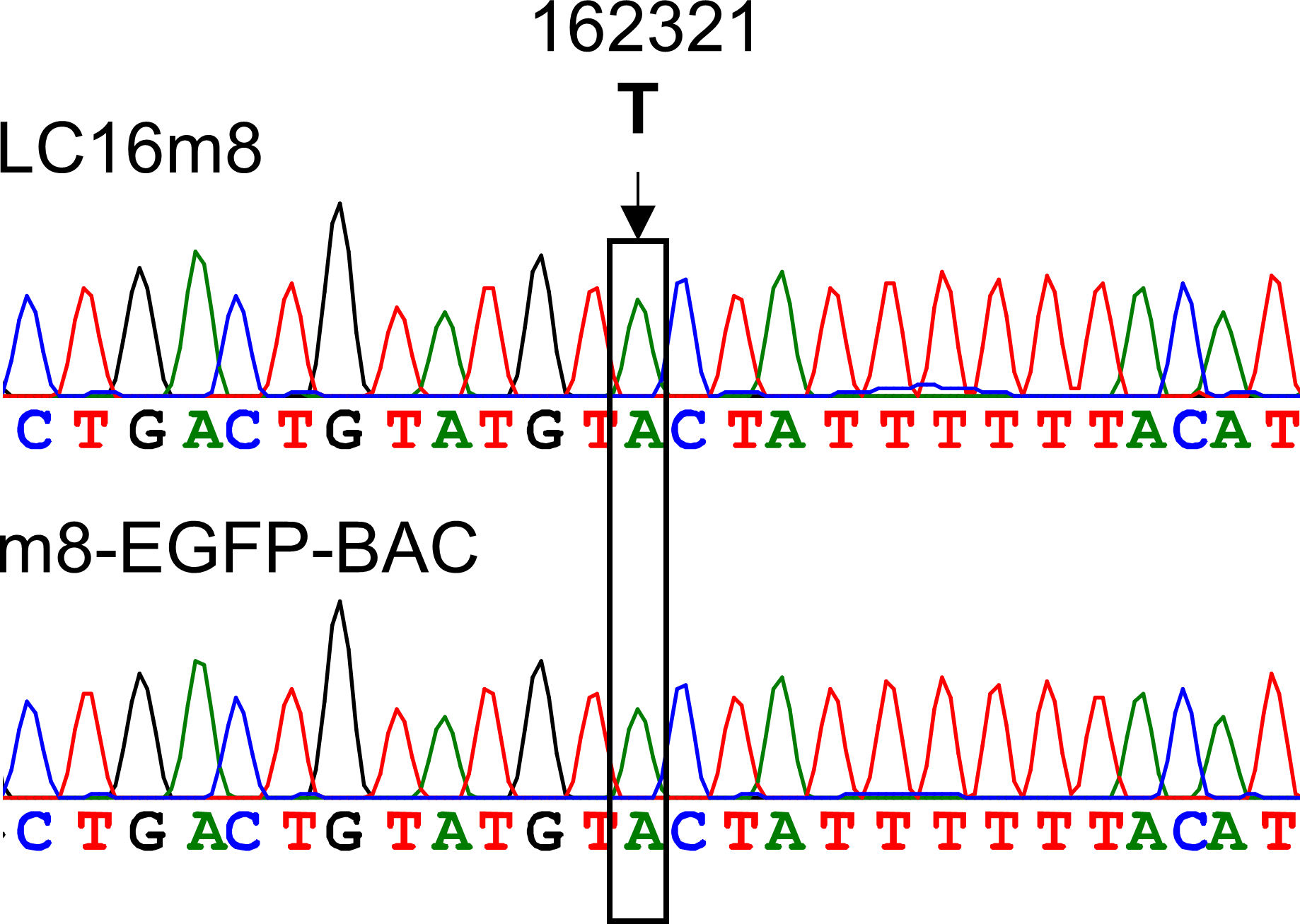

Supplement: S2 Fig — The chromatograms of m8 and m8-EGFP-BAC around position 162321 are shown. (TIF) [file pone.0192725.s003.tif]

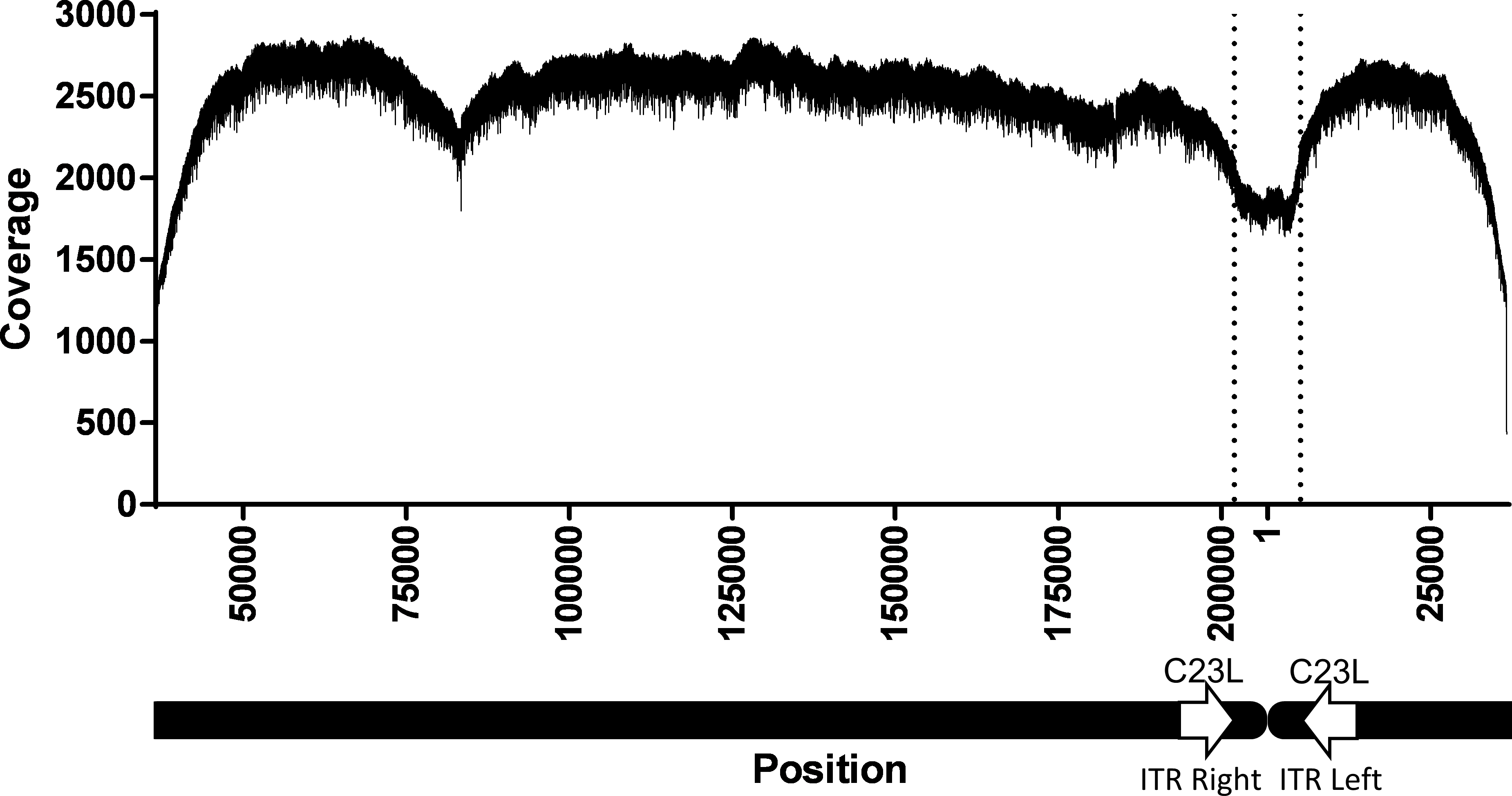

Supplement: S3 Fig — (TIF) [file pone.0192725.s004.tif]

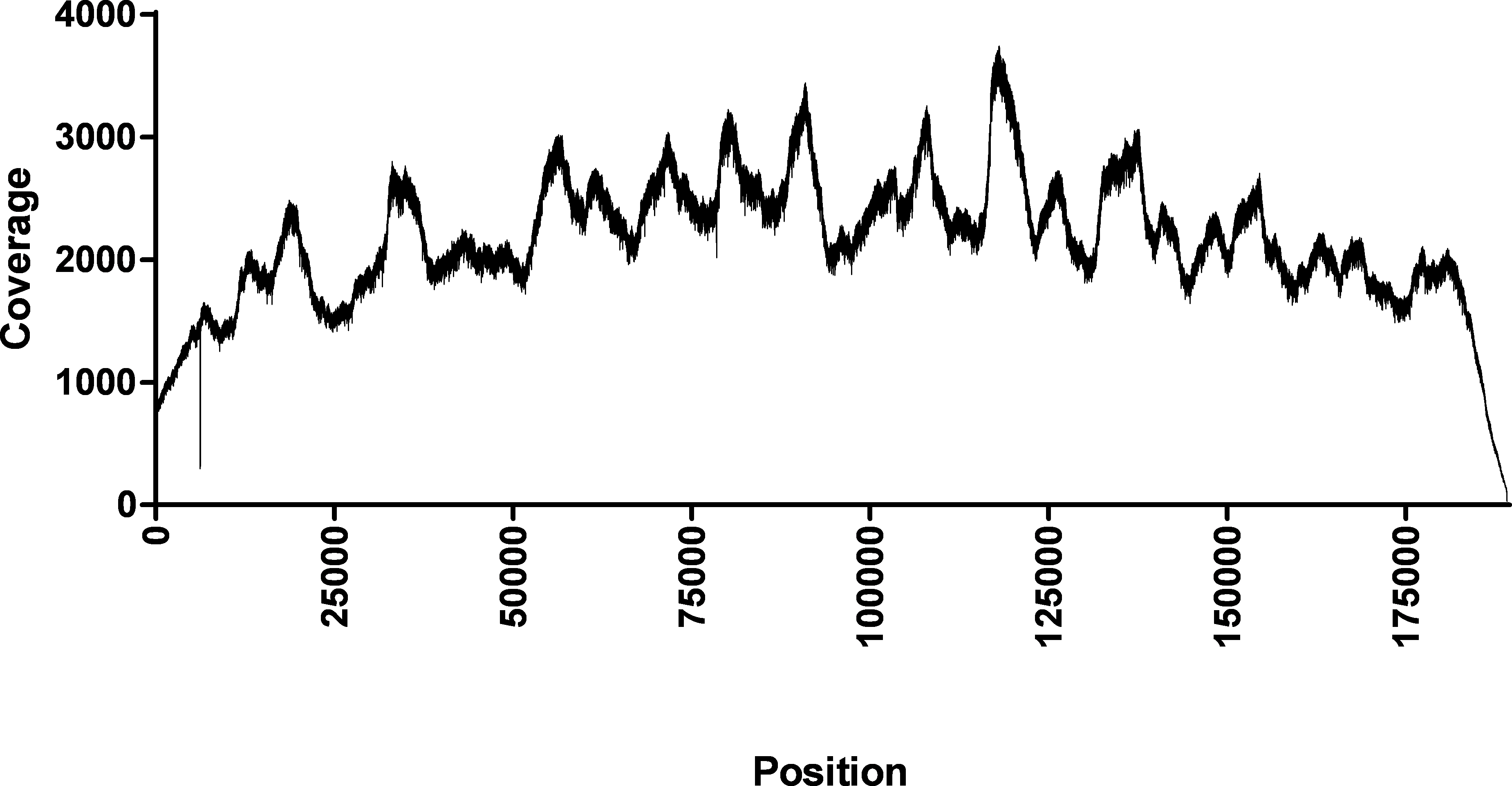

Supplement: S4 Fig — (TIF) [file pone.0192725.s005.tif]

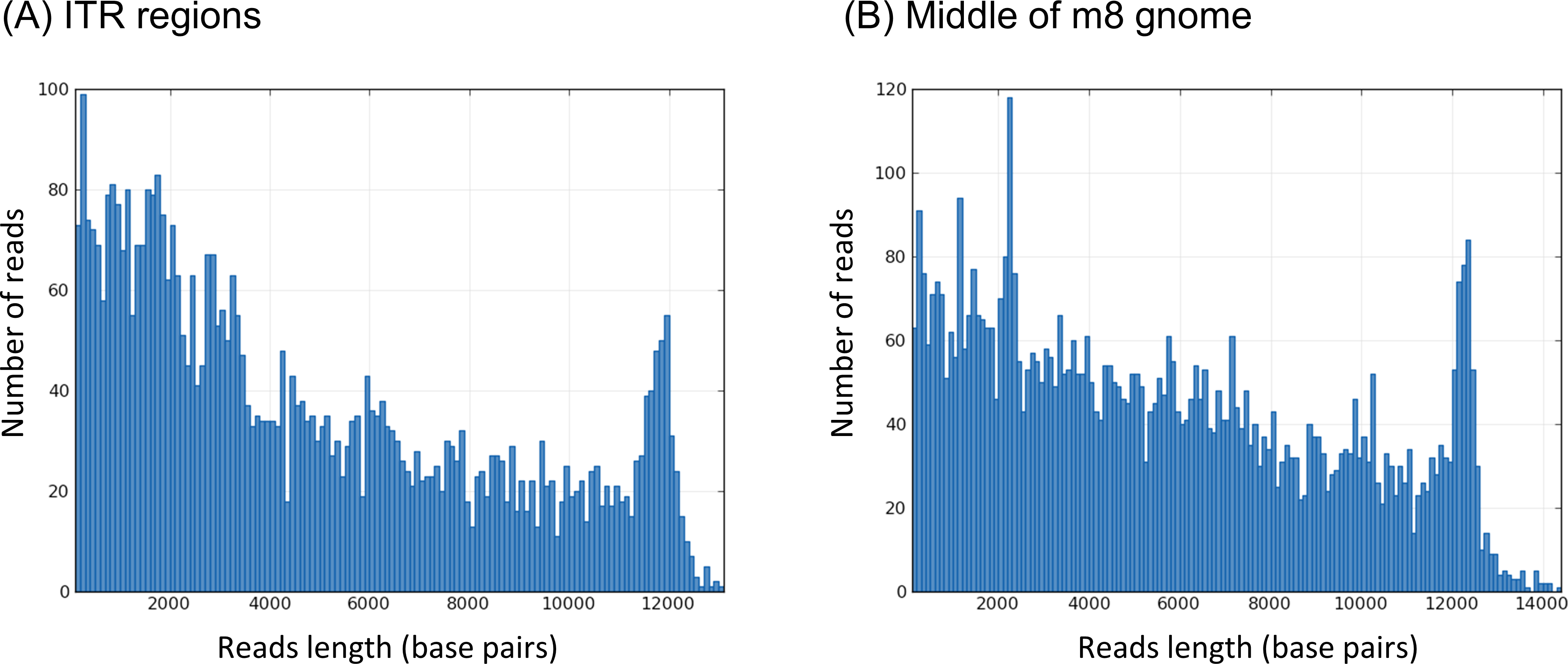

Supplement: S5 Fig — The region is flanked by C23L genes (A) and the middle of the m8 genome at position 80000 to 92000 (B). (TIF) [file pone.0192725.s006.tif]
